# Hypothalamic mTORC2 is essential for metabolic health and longevity

**DOI:** 10.1111/acel.13014

**Published:** 2019-08-01

**Authors:** Karthikeyani Chellappa, Jacqueline A. Brinkman, Sarmistha Mukherjee, Mark Morrison, Mohammed I. Alotaibi, Kathryn A. Carbajal, Amber L. Alhadeff, Isaac J. Perron, Rebecca Yao, Cole S. Purdy, Denise M. DeFelice, Matthew H. Wakai, Jay Tomasiewicz, Amy Lin, Emma Meyer, Yajing Peng, Sebastian I. Arriola Apelo, Luigi Puglielli, J. Nicholas Betley, Georgios K. Paschos, Joseph A. Baur, Dudley W. Lamming

**Affiliations:** ^1^ Department of Physiology and Institute for Diabetes, Obesity and Metabolism, Perelman School of Medicine University of Pennsylvania Philadelphia PA USA; ^2^ Department of Medicine University of Wisconsin‐Madison Madison WI USA; ^3^ William S. Middleton Memorial Veterans Hospital Madison WI USA; ^4^ Endocrinology and Reproductive Physiology Graduate Training Program University of Wisconsin‐Madison Madison WI USA; ^5^ Department of Biology, School of Arts and Sciences University of Pennsylvania Philadelphia PA USA; ^6^ Center for Sleep and Circadian Neurobiology, Perelman School of Medicine University of Pennsylvania Philadelphia PA USA; ^7^ Department of Dairy Science University of Wisconsin‐Madison Madison WI USA; ^8^ Waisman Center University of Wisconsin‐Madison Madison WI USA; ^9^ The Institute for Translational Medicine and Therapeutics, Perelman School of Medicine University of Pennsylvania Philadelphia PA USA

**Keywords:** frailty, hypothalamus, mTOR, mTORC2, lifespanobesity, obesity

## Abstract

The mechanistic target of rapamycin (mTOR) is an evolutionarily conserved protein kinase that regulates growth and metabolism. mTOR is found in two protein complexes, mTORC1 and mTORC2, that have distinct components and substrates and are both inhibited by rapamycin, a macrolide drug that robustly extends lifespan in multiple species including worms and mice. Although the beneficial effect of rapamycin on longevity is generally attributed to reduced mTORC1 signaling, disruption of mTORC2 signaling can also influence the longevity of worms, either positively or negatively depending on the temperature and food source. Here, we show that loss of hypothalamic mTORC2 signaling in mice decreases activity level, increases the set point for adiposity, and renders the animals susceptible to diet‐induced obesity. Hypothalamic mTORC2 signaling normally increases with age, and mice lacking this pathway display higher fat mass and impaired glucose homeostasis throughout life, become more frail with age, and have decreased overall survival. We conclude that hypothalamic mTORC2 is essential for the normal metabolic health, fitness, and lifespan of mice. Our results have implications for the use of mTORC2‐inhibiting pharmaceuticals in the treatment of brain cancer and diseases of aging.

## 1 INTRODUCTION

The mechanistic target of rapamycin (mTOR) is a serine/threonine kinase that plays critical roles in the regulation of growth, metabolism, and aging. The mTOR protein kinase is found in two distinct protein complexes; mTOR complex 1 (mTORC1) integrates numerous environmental and hormonal cues, including the availability of amino acids (Wolfson & Sabatini, 2017), to regulate key anabolic processes including ribosomal biogenesis, protein translation, and autophagy, while mTOR complex 2 (mTORC2) plays a role in cytoskeletal organization and is a key effector of insulin/PI3K signaling (Kennedy & Lamming, 2016; Zhou & Huang, 2011). The pharmaceutical rapamycin, which acutely and robustly inhibits mTORC1, extends the lifespan in organisms including yeast, worms, flies, and mice, even when begun late in life or when treatment is intermittent (Apelo, Pumper, Baar, Cummings, & Lamming, 2016; Arriola Apelo & Lamming, 2016; Bitto et al., 2016; Bjedov et al., 2010; Dumas & Lamming, 2019; Hansen et al., 2007; Harrison et al., 2009; Kapahi et al., 2004; Miller et al., 2011; Powers, Kaeberlein, Caldwell, Kennedy, & Fields, 2006; Robida‐Stubbs et al., 2012; Selman et al., 2009).

While it has long been presumed that inhibition of mTORC1 by rapamycin mediates its beneficial effects on longevity, we and others have found that prolonged treatment with rapamycin also inhibits mTORC2, both in cell culture and in vivo in mice (Lamming et al., 2012; Sarbassov et al., 2004; Schreiber et al., 2015). However, inhibition of mTORC2 by rapamycin is limited to specific cell lines and tissues, most likely determined by the relative expression of FK506‐binding proteins (FKBPs), FKBP12 and FKBP51 (Schreiber et al., 2015). In the nematode *Caenorhabditis elegans*, mTORC2 regulates metabolic processes via several distinct signaling pathways and can have positive or negative effects on lifespan depending on the tissue that is targeted, the temperature, and the food source (Mizunuma, Neumann‐Haefelin, Moroz, Li, & Blackwell, 2014; Robida‐Stubbs et al., 2012; Soukas, Kane, Carr, Melo, & Ruvkun, 2009). In mice, disruption of mTORC2 signaling via deletion of *Rictor*, which encodes an essential protein component, in the liver, adipose tissue, or skeletal muscle leads to insulin resistance (Bentzinger et al., 2008; Kumar et al., 2008, 2010; Lamming, Demirkan, et al., 2014; Lamming, Mihaylova, et al., 2014; Polak et al., 2008; Tang et al., 2016). We also recently showed that deletion of hepatic *Rictor*, or postdevelopmental depletion of RICTOR in the whole body of mice, significantly reduced male lifespan (Lamming, Mihaylova, et al., 2014).

Over the last decade, significant progress has been made in understanding the roles of both mTOR complexes in the regulation of key metabolic tissues (Kennedy & Lamming, 2016). Less well understood is the role of mTOR complex signaling in the brain. mTOR Complex 1 is clearly an important regulator of neuronal behavior; hypothalamic mTORC1 is a key sensor of nutrient sufficiency and acute activation of hypothalamic mTORC1 suppresses food intake, while chronic activation selectively in POMC neurons can drive overnutrition and obesity (Cota et al., 2006; Mori et al., 2009; Yang et al., 2012). Genetic reduction of *S6K1*, a key downstream effector of mTORC1, or prophylactic treatment with rapamycin, which can cross the blood‐brain barrier (Cloughesy et al., 2008; Gottschalk et al., 2011) delays or prevents the progression of Alzheimer's disease in mouse models (Caccamo et al., 2015; Majumder et al., 2012; Spilman et al., 2010) and also blocks age‐associated cognitive decline in wild‐type mice (Halloran et al., 2012). In contrast, the role of brain mTORC2 signaling in the regulation of metabolism, health, and longevity has been less studied. This knowledge gap has recently begun to narrow, with recent work showing that deletion of *Rictor* in male mice using the neuron‐specific Nestin‐Cre recombinase decreases energy expenditure and increases adiposity without affecting food intake, lowers body temperature, and disrupts glucose homeostasis (Kocalis et al., 2014). Body weight was also affected in male mice by selective loss of *Rictor* in POMC neurons, but in this case, the primary effect was on food intake rather than energy expenditure (Kocalis et al., 2014). Thus, mTORC2 signaling in the brain plays important roles in whole body metabolism, but the specific neuronal populations mediating these effects and the long‐term implications for health and longevity remain to be elucidated.

In order to further our understanding of where and when neuronal mTORC2 might be important, we examined the phosphorylation of its substrate AKT S473 in the brains of both male and female mice across their lifespans. We determined that neuronal mTORC2 signaling increases with age in distinct brain regions including the hypothalamus. In order to elucidate the roles of hypothalamic mTORC2 in the metabolic health and aging of mice, we created a model (*Rictor^Nkx2.1−/−^*) in which *Rictor* is deleted in a wide range of hypothalamic neurons using *Nkx2.1‐Cre*. We find that *Rictor^Nkx2.1−/−^* mice of both sexes exhibit lifelong increases in adiposity starting at an early age and have reduced spontaneous locomotor activity. *Rictor^Nkx2.1−/−^* mice have decreased glucose tolerance, develop insulin resistance as they age, display increased frailty, and ultimately have a reduced lifespan. Finally, we find that *Rictor^Nkx2.1−/−^* mice have increased susceptibility to diet‐induced obesity. Our results demonstrate a key role for hypothalamic mTORC2 in the regulation of metabolism, fitness, and longevity, and suggest that inhibition of this complex by pharmaceuticals must be approached with caution.

## 2 RESULTS

### 2.1 mTORC2 signaling increases with age in hypothalamic neurons

**Figure 1:**
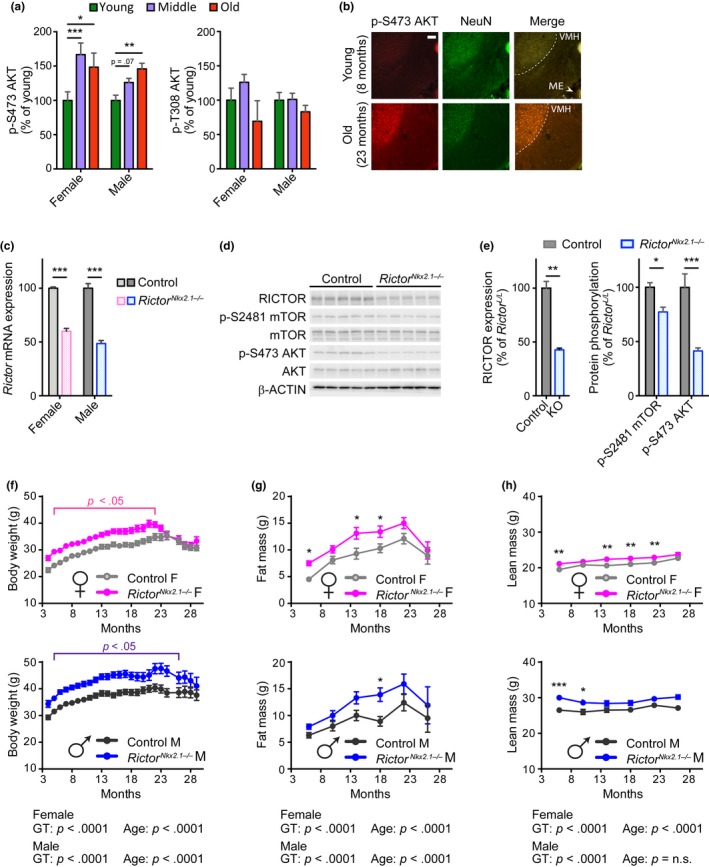
Hypothalamic mTORC2 signaling increases with age and regulates body weight homeostasis. (a) Quantification of phosphorylated AKT residues in whole brain lysate from fasted female and male C57BL.6J.Nia mice; young refers to 6‐month‐old males and females (10 males, 5 females), middle refers to 24‐month‐old males and 22‐month‐old females (10 males, 5 females), and old refers to 30‐month‐old males and 26‐month‐old females (8 males, 4 females). Quantification of phosphorylated proteins are relative to total protein (Dunnett's test following two‐way ANOVA, * = *p < *.05, ** = *p < *.01, *** = *p < *.001). (b) mTORC2 activity, as determined by IHC‐IF for phosphorylated Akt S473 (in red), is increased in the hypothalamus of overnight fasted 23‐month‐old female C57BL.6J.Nia mice relative to young 8‐month‐old mice. A neuronal nuclei marker is targeted by the NeuN antibody (in green), showing the mTORC2 signaling is increased in aged neurons in these regions. Shown are representative images of hypothalamic regions (total *n* examined = 4 mice/group). Scale bar = 100 µm. (c) Expression of *Rictor* mRNA in hypothalamic tissue of 3‐ to 6‐month‐old *Rictor^Nkx2.1−/−^* mice and controls (*n* = 5‐8/group; *** = *p < *.001, Holm–Sidak test following two‐way ANOVA). (d) Hypothalamic protein lysates from 6‐month‐old male control and *Rictor^Nkx2.1−/−^* mice were immunoblotted for phosphorylated and total AKT, phosphorylated and total mTOR, RICTOR, and β‐ACTIN. (e) Quantification of RICTOR expression relative to β‐ACTIN and phosphorylated mTOR and AKT relative to total protein (*n* = 5 control and 9 *Rictor^Nkx2.1−/−^* mice; left: ** = *p < *.01, *t* test; right: Holm–Sidak test following two‐way ANOVA, * = *p < *.05, *** = *p < *.001). (f) Longitudinal assessment of body weight of control and *Rictor^Nkx2.1−/−^* mice (*n* = 5–35 per group; *p < *.05 indicates significant difference between genotypes at each time point within the indicated range, Holm–Sidak test following two‐way ANOVA). (g, h) Longitudinal assessment of (g) fat mass and (H) lean mass of control and *Rictor^Nkx2.1−/−^* mice (*n* = 5–29 mice/group; Holm–Sidak test following two‐way ANOVA, * = *p < *.05, ** = *p < *.01, *** = *p < *.001). (f–h) The overall effect of genotype (GT), age, and the interaction represents the *p*‐value from a two‐way ANOVA. Error bars represent the *SEM*

We studied brains from three different age‐groups of C57BL/6J.Nia mice obtained from the NIA Aged Rodent Colony: a “young” group, aged 6 months; a “middle” group of 22‐month‐old females and 24‐month‐old males (approximately 70% survival for each sex, based on published lifespan curves for C57BL/6J.Nia mice (Turturro et al., 1999)); and an “old” group of 26‐month‐old females and 30‐month‐old males (approximately 30% survival). We observed increased phosphorylation of the mTORC2 target AKT S473 in whole brain lysates from 22‐ and 26‐month‐old females and 30‐month‐old males relative to young control mice (Figure 1a and Figure S1a). This effect was specific to mTORC2 and not representative of a generalized increase in insulin/IGF‐1 signaling, as phosphorylation of AKT T308, an mTORC2‐independent site downstream of insulin signaling, was not increased in aged mice of either sex. In order to identify the specific regions of the brain that contributed to the increased mTORC2 signaling, we performed immunohistochemistry with antibodies against phosphorylated AKT S473 and NeuN, a marker of neuronal nuclei. We found that phosphorylation of AKT S473 increased in specific regions of the aged mouse brain, including the neurons of the hypothalamus as well as cells within the cortex and thalamus (Figure 1b, Figure S1b).

### 2.2 Development of mice lacking *Rictor* in hypothalamic neurons

Mice lacking mTORC2 signaling in hypothalamic neurons were generated by crossing mice conditionally expressing *Rictor* to mice expressing Cre recombinase under the control of the *Nkx2.1* promoter (*Rictor^Nkx2.1−/−^*) (Shiota, Woo, Lindner, Shelton, & Magnuson, 2006; Xu, Tam, & Anderson, 2008). This promoter is active in most of the hypothalamic nuclei during early development, with the exception of the suprachiasmatic nucleus (Mieda, Hasegawa, Kessaris, & Sakurai, 2017; Ring & Zeltser, 2010; Xu et al., 2008). We verified deletion of *Rictor* in the hypothalamus by determining the expression of *Rictor* mRNA and RICTOR protein from both male and female mice (Figure 1c–e and Figure S1c). mTORC2 activity was assessed by determining phosphorylation of the mTORC2 substrate AKT S473, as well as phosphorylation of mTOR itself at S2481, an autophosphorylation site associated with incorporation into mTORC2 (Copp, Manning, & Hunter, 2009). As expected, the phosphorylation of AKT S473 and mTOR S2481 was reduced in *Rictor^Nkx2.1−/−^* mice. Brain size was normal in *Rictor^Nkx2.1−/−^* mice with no gross abnormalities apparent (Figure S1d).

### 2.3 Early‐onset, lifelong increase in body weight and adiposity of *Rictor^Nkx2.1−/−^* mice

We monitored the body weight and composition of *Rictor^Nkx2.1−/−^* mice and their wild‐type littermates. We observed that mice lacking hypothalamic *Rictor* weighed more than their wild‐type littermates throughout their lifespan, a difference that was statistically significant up to 22 months of age in females and 26 months of age in males (Figure 1f). Periodic assessment of body composition demonstrated that in the aging cohort of *Rictor^Nkx2.1−/−^* mice, both sexes had increases in fat mass (Figure 1g) and to a lesser extent, lean mass (Figure 1h); the overall effect was a lifelong increase in adiposity (Figure S1e,f).

**Figure 2:**
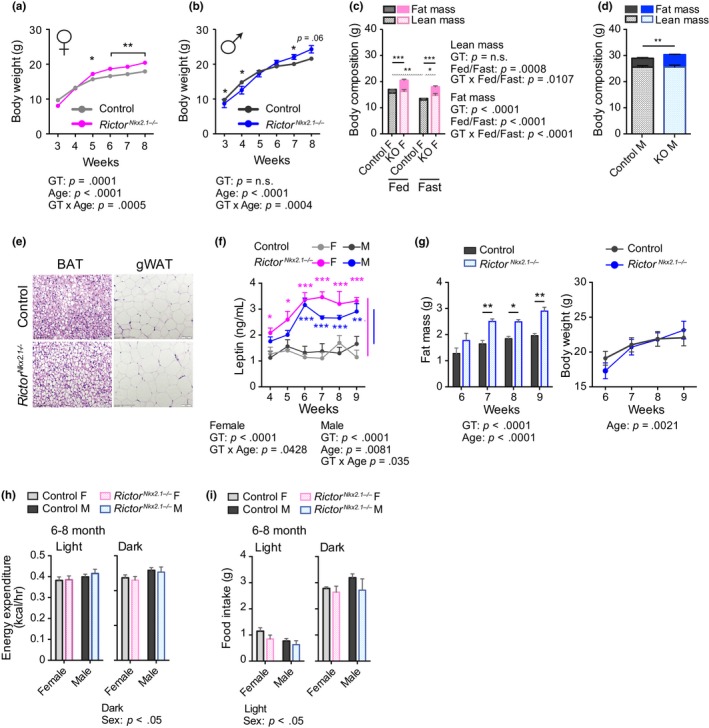
Early onset of obesity in mice lacking *Rictor* in hypothalamic neurons. (a and b) The weights of (a) female and (b) male control and *Rictor^Nkx2.1−/−^* mice were tracked from 3 to 8 weeks of age (*n* varies by time point and group, *n* = 4–31; Holm–Sidak test following two‐way ANOVA, * = *p < *.05, ** = *p < *.01). (c and d) Lean and fat mass in (c) 10‐wk‐old female mice (*n* = 5‐6/group; Holm–Sidak test following two‐way ANOVA, * = *p < *.05, ** = *p < *.01, *** = *p < *.001, solid lines indicate comparisons of fat mass and spotted lines indicate comparison of lean mass) and (d) 20‐ to 22‐week‐old male mice (*n* = 5‐9/group; *t* test, ** = *p < *.01). (e) H&E‐stained BAT and gonadal white adipose tissue from 24‐ to 26‐wk‐old chow fed male mice. (f) Plasma leptin levels of female and male *Rictor^Nkx2.1−/−^* mice (*n* = 6–8 mice/group; Sidak test following two‐way ANOVA, * = *p < *.05, ** = *p < *.01, *** = *p < *.001, blue/pink stars indicate significant difference vs. male/female controls). (Corresponding body weight curve is represented in Figure 6a, week four to nine on chow diet) (g) Fat mass (Left) and body weight (Right) of male control and *Rictor^Nkx2.1−/−^* mice (*n* = 5–6 mice/group; Sidak test following two‐way ANOVA, * = *p < *.05, *** = *p < *.001). (h) Energy expenditure of 24‐ to 33‐wk‐old female and male mice; per mouse basis (*n* = 6 mice/group; Sidak test following two‐way ANOVA, * = *p < *.05). (i) Twenty‐four hour food intake of 24‐ to 33‐wk‐old female and male mice on normal chow (*n* = 6 mice/group; Sidak test following two‐way ANOVA, * = *p < *.05).The overall effect of either genotype (GT) and age (panels A, B, F, G), GT and feeding status (C) or GT and sex (panels H‐I), and the interaction represents the *p*‐value from a two‐way ANOVA. Error bars represent the *SEM*

To characterize the development of these body weight and composition phenotypes, we analyzed a second cohort of *Rictor^Nkx2.1−/−^* mice and their wild‐type littermates from the time of weaning. *Rictor^Nkx2.1−/−^* mice of both sexes tended to be lighter than littermate controls at weaning, an effect that was statistically significant in males, yet by 5–7 weeks of age *Rictor^Nkx2.1−/−^* mice of both sexes weighed more than littermate controls (Figure 2a,b). Weight gain generally occurred over a distinct period with subsequent stabilization at a new set point relative to controls. Increased body weight in young *Rictor^Nkx2.1−/−^* mice reflected an increase in fat mass without any change in lean mass (Figure 2c,d). Consistently, fat pads were heavier in *Rictor^Nkx2.1−/−^* mice (Figure S2a,b), and larger lipid droplets and adipocyte hypertrophy were observed in brown and white adipose tissue, respectively (Figure 2e).

Leptin is an adipose‐derived hormone that decreases food intake and promotes energy expenditure (Campbell et al., 2017; Chua et al., 1996; Halaas et al., 1995; Pelleymounter et al., 1995). Loss of leptin signaling is sufficient to cause drastic weight gain, whereas weight gain due to other mechanisms is associated with hyperleptinemia as a compensatory response. As *Rictor^Nkx2.1−/−^* mice have increased adiposity, we determined leptin levels in knockouts and their wild‐type littermates. We observed that plasma leptin was significantly increased in *Rictor^Nkx2.1−/−^* mice, as was leptin mRNA expression in adipose tissue (Figure 2f and Figure S2c). Intriguingly, the increase in plasma leptin was observed in *Rictor^Nkx2.1−/−^* mice prior to a measurable increase in body weight or adipose mass (Figure 2f,g), and without obvious differences in adipocyte size (Figure S2d,e). Together, our results suggest that mTORC2 signaling in Nkx2.1 neurons may have primary effects on leptin expression independent of adipose mass.

### 2.4 Food intake and energy expenditure in *Rictor^Nkx2.1−/−^* mice

We did not detect significant differences in body weight, respiratory exchange ratio (RER), food intake, or locomotor activity in 4‐week‐old *Rictor^Nkx2.1−/−^* mice (Figure S2f–i). Although *Rictor^Nkx2.1−/−^* mice displayed a slight decrease in energy expenditure on a per animal basis, the effect was not significant after correcting for the slightly lower body weight of the female *Rictor^Nkx2.1−/−^* mice at this age, either by dividing energy expenditure by mass or by using ANCOVA (Figure S2j and Table S1). While no consistent change in food intake was detected during the period of body weight gain (Figure S2k), we did note a trend toward increased hyperphagia in male *Rictor^Nkx2.1−/−^* mice upon refeeding (Figure S2l), indicating some dysregulation of the mechanism controlling satiety. In adult (24–33 week‐old) mice, body weight was higher in *Rictor^Nkx2.1−/−^* mice of both sexes than in their wild‐type littermates (Figure S2m), while average energy expenditure per mouse and food intake were not affected by genotype in either gender (Figure 2h,i). Body mass adjusted energy expenditure (ANCOVA) was reduced in adult female *Rictor^Nkx2.1−/−^* mice relative to their wild‐type littermates, which is consistent with their increased adiposity since adipose tissue consumes less energy per unit mass (Table S1). At ten months of age, opposite trends were observed in the energy expenditure between genders; total energy expenditure per mouse was slightly increased in females and slightly reduced in male *Rictor^Nkx2.1−/−^* mice. The effect in females was absent after dividing energy expenditure by body weight, but remained marginally significant when adjusting by ANCOVA. Thus, energy expenditure is unchanged or increased in females, suggesting that food intake must explain the higher body weight. In contrast, the decreased energy expenditure in males occurs despite their larger size and unchanged food intake, suggesting that decreased calorie output might contribute to the maintenance of higher body mass in this sex (Figure S3a–c and Table S1). However, neither food intake nor daily energy expenditure were significantly changed in subsequent measures (at 18 months of age) from the same *Rictor^Nkx2.1−/−^* mice, with or without adjustment for body weight by ANCOVA (Figure S4a–c and Table S1). Intriguingly, beam break analysis revealed decreased locomotor activity in 6‐ and 10‐month‐old *Rictor^Nkx2.1−/−^* mice (Figure S5a–c), a phenotype that has not been previously associated with the mTORC2 signaling pathway.

### 2.5 *Rictor^Nkx2.1−/−^* mice are hypoactive and exhibit reduced voluntary activity

**Figure 3:**
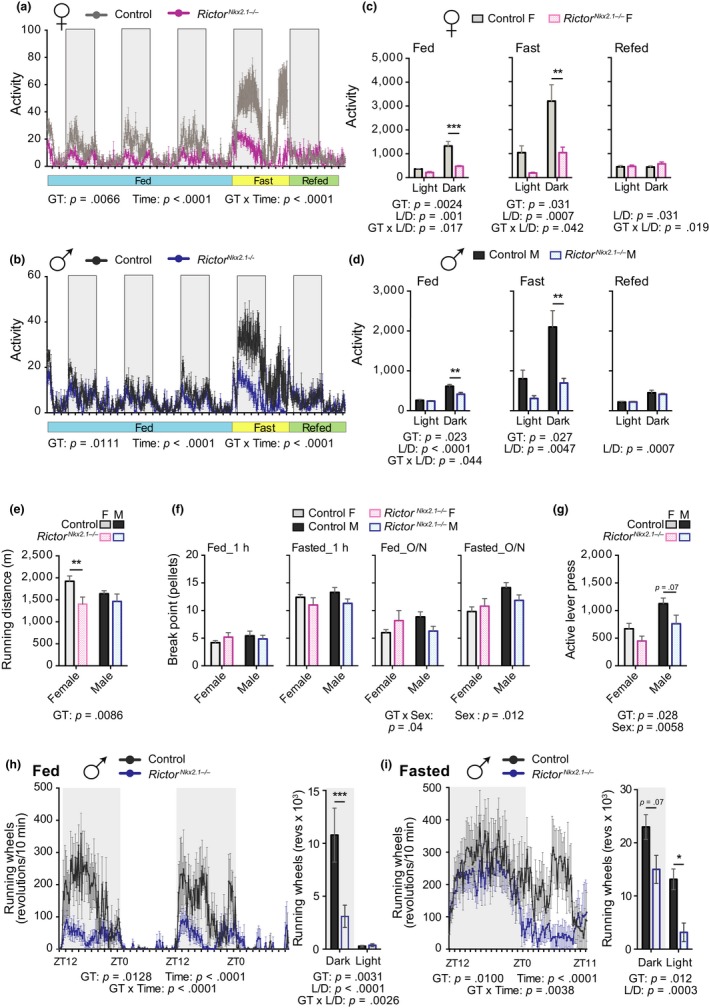
Voluntary home cage and running wheel activity but not goal‐oriented tasks is reduced in *Rictor^Nkx2.1−/−^* mice. (a and b) Traces of average home cage activity of 13‐ to 14‐wk‐old female (a) and male (b) mice under the conditions indicated, as determined by telemetry with counts binned into 10‐min blocks. The overall effect of genotype (GT), time, and the interaction represents the *p*‐value from a two‐way RM ANOVA. (c and d) Quantification of the data in panels a and b; activity during the fed condition represents a two day average; during fasting and refeeding over ~24‐hr time period (*n* = 4–5 mice/group; Sidak test following two‐way ANOVA, * = *p < *.05, ** = *p < *.01, *** = *p < *.001). (e) Average distance run on a treadmill at exhaustion for 11‐wk‐old male and female mice (*n* = 4–10 mice/group, Sidak test following two‐way ANOVA, * = *p < *.05, ** = *p < *.01). (f) Eight‐month‐old control and *Rictor^Nkx2.1−/−^* mice of both sexes were trained to press a lever to obtain food pellets. Pellets received prior to a 10‐min gap without earning a pellet (the “break point”) in a progressive ratio operant task conducted for one hour during the light period under fed and fasted conditions (1h PR) or during an overnight progressive ratio operant task under fed and fasted conditions (overnight PR). (*n* = 5–7 mice/group; Sidak test following two‐way ANOVA, * = *p < *.05). (g) Number of active lever presses during an overnight extinction paradigm where mice do not receive food pellets in response to lever presses (*n* = 5–7 mice/group; Sidak test following two‐way ANOVA, * = *p < *.05). (e–g) The overall effect of genotype (GT), sex, and the interaction represents the *p*‐value from a two‐way ANOVA. (h and i) Voluntary running wheel activity of 4‐month‐old male mice during (h) ad libitum feeding and (i) 24‐hr food deprivation. Data represented as revolutions per 10‐min bin. Inset, cumulative running wheel activity during the light and dark periods (*n* = 5‐8/group; Sidak test following two‐way RM ANOVA, * = *p < *.05, *** = *p < *.001). (h and i) The overall effect of genotype (GT), time, and the interaction represents the *p*‐value from a two‐way ANOVA. Error bars represent the *SEM*

To more definitively assess spontaneous locomotor activity in *Rictor^Nkx2.1−/−^* mice in their home cage environment, we employed telemetry. We found that activity was robustly decreased in both sexes, although the effect was most pronounced in females (Figure 3a–d), owing in part to the fact that wild‐type females are considerably more active than their male counterparts. Fasting markedly increased locomotor activity, as expected (Yamanaka et al., 2003), but the decreased activity phenotype remained in *Rictor^Nkx2.1−/−^* mice. Body weight and locomotor activity were not correlated in this experiment, suggesting that the phenotype was not secondary to changes in body weight per se (Figure S5d).

A neural circuit involving the preoptic area and dorsomedial hypothalamus was recently shown to influence both physical activity and core body temperature (Zhao et al., 2017), and mice lacking *Rictor* in the whole brain have decreased core body temperature (Kocalis et al., 2014). Using implanted telemetry probes, we observed a subtle but consistent reduction in core body temperature in ad libitum fed female *Rictor^Nkx2.1−/−^* mice during the middle of the dark period, with a similar tendency that did not reach statistical significance in males (Figure S6a–d).

In rodents, locomotor activity can be categorized as spontaneous (e.g., voluntary activity or exploration) or motivated (e.g., food seeking). To first establish that the decreased locomotor activity in *Rictor^Nkx2.1−/−^* mice was not due to motor deficits, we tested running using an accelerating treadmill protocol (Frederick et al., 2015). Both sexes of *Rictor^Nkx2.1−/−^* mice were able to run (Figure 3e), exhibiting activity levels far in excess of spontaneous home cage movement (Majdak et al., 2016; Zombeck, Deyoung, Brzezinska, & Rhodes, 2011). We observed an overall effect of genotype on the total distance run at exhaustion, with a small but significant reduction for female *Rictor^Nkx2.1−/−^* mice (Figure 3e). This small effect is not consistent with a major motor deficit, and may be attributable to the increased body weight of mice lacking hypothalamic *Rictor*, and/or their decreased habitual level of activity.

To address the possibility of altered food seeking behavior in *Rictor^Nkx2.1−/−^* mice, we assessed their motivation to obtain food. Mice were trained to press a lever to obtain a food pellet, then subjected to a progressive ratio (PR) schedule of reinforcement, where the number of lever presses required to obtain each food pellet increased exponentially (Alhadeff & Grill, 2014; Betley et al., 2015). Break point analysis (number of pellets received prior to a 10‐min break in lever pressing) revealed that control and *Rictor^Nkx2.1−/−^* mice are equally motivated to obtain food under both fed and fasted conditions in this operant task, whether assessed for one hour during the light period or overnight during the dark period (Figure 3f). Next, we determined the willingness of *Rictor^Nkx2.1−/−^*mice to press the lever in the absence of a food pellet reward (extinction) and observed no significant effects (Figure 3g). Together, these data indicate that *Rictor^Nkx2.1−/−^* mice have no change in motivation to obtain food, which suggests that goal‐oriented activity is retained in *Rictor^Nkx2.1−/−^* mice despite altered basal activity (Krashes et al., 2011). We next placed running wheels in home cages to assess the intrinsic motivation of *Rictor^Nkx2.1−/−^* mice toward physical activity. *Rictor^Nkx2.1−/−^* mice exhibited a major decrease in voluntary wheel running when fed ad libitum (Figure 3h). Upon food deprivation, running wheel activity was partly restored during the dark phase in *Rictor^Nkx2.1−/−^* mice (Figure 3i), but the expected increase in activity during the light period relative to ad libitum fed mice was largely blunted (Krizo et al., 2018). These data support the conclusion that *Rictor^Nkx2.1−/−^* mice have a reduced intrinsic drive to be physically active.

### 2.6 Assessment of endocrine, hormonal and neuropeptide changes in *Rictor^Nkx2.1−/−^* mice

As Nkx2.1‐Cre has been shown to be active not only in the hypothalamus, but also in cells within the thyroid and pituitary (Xu et al., 2008), we next assessed whether *Rictor^Nkx2.1−/−^* mice displayed changes in related pathways that might contribute to their growth and body weight phenotypes. Intriguingly, we found a significant increase in the circulating level of insulin‐like growth factor 1 (IGF‐1) in both sexes of *Rictor^Nkx2.1−/−^* mice relative to their wild‐type littermates (Figure S7a). Consistent with increased growth hormone/IGF‐1 action, we observed a statistically significant increase in the femur lengths of female *Rictor^Nkx2.1−/−^* mice (Figure S7b) as well as increases in the weights of lean tissues, including liver and skeletal muscle, in three‐month‐old mice (Figure S7c,d). In contrast, we found no significant differences in T4 or corticosterone levels between *Rictor^Nkx2.1−/−^* knockouts and their littermate controls (Figure S7e,f). Thus, we consider it unlikely that the metabolic phenotypes we observe are a result of altered mTORC2 activity in the thyroid or changes in the hypothalamus–pituitary–adrenal axis, but increased IGF‐1 signaling may play a role in the increased lean mass. Notably, growth hormone/IGF‐1 signaling is normally associated with decreased adiposity (Bengtsson et al., 1993; Berryman, Glad, List, & Johannsson, 2013) and thus cannot explain the expanded adipose tissue mass in *Rictor^Nkx2.1−/−^* mice.

The fact that *Rictor^Nkx2.1−/−^* mice maintain increased adiposity despite high leptin is consistent with the possibility that they are leptin resistant. Since many leptin‐responsive neurons are located within the hypothalamus, one possibility is that mTORC2 is directly required downstream of leptin to suppress food intake. To test this possibility, we injected control and knockout mice with recombinant leptin and found that high‐dose leptin suppresses food intake and body weight in *Rictor^Nkx2.1−/−^* mice (Figure S8a,b). We also observed similar levels of pSTAT3 in the hypothalamus of control and *Rictor^Nkx2.1−/−^* mice (Figure S8c,d), suggesting that proximal leptin signaling is at normal levels. To further probe potential mechanisms underlying the dysregulated body weight in *Rictor^Nkx2.1−/−^* mice, we examined the expression of hypothalamic neuropeptides. The orexigenic neuropeptide NPY was significantly increased in females, whereas the anorexigenic POMC and CART were reduced by ~50% and 25%, respectively, in males (Figure S8e,f). Thus, *Rictor* deletion in Nkx2.1‐expressing neurons altered the expression of several hypothalamic neuropeptides known to influence satiety and food intake.

### 2.7 mTORC2 signaling is essential for healthspan and lifespan

**Figure 4:**
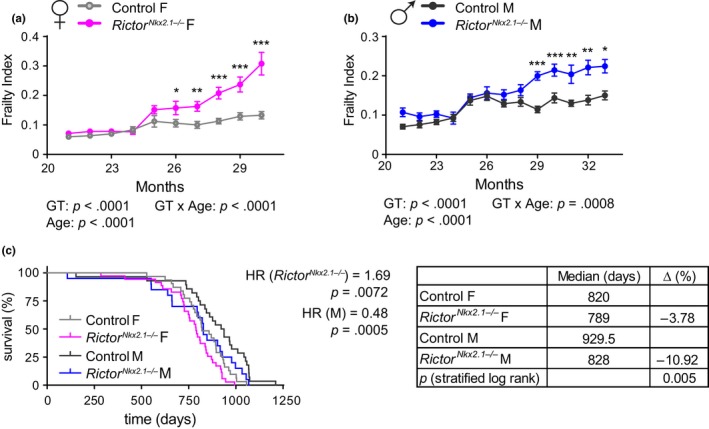
Hypothalamic mTORC2 signaling is essential for healthspan and lifespan. (a and b) Frailty was assessed longitudinally in (a) female and (b) male mice starting at 21 months of age (*n* = numbers vary month by month; 2–24 mice/group at each time point). (c) Kaplan–Meier plot showing the lifespan of female and male control and *Rictor^Nkx2.1−/−^* mice. The overall effect of genotype (*Rictor^Nkx2.1−/−^*) and sex (M) was determined using a Cox proportional hazards test (HR, hazard ratio). The table shows the median lifespan for each group, the percentage decrease in median lifespan for each sex, and the two‐tailed stratified log‐rank *p*‐value for the decrease in lifespan as a result of deletion of hypothalamic *Rictor*. Error bars represent the *SEM*

We next sought to determine the overall effect of hypothalamic *Rictor* deletion on health and longevity. Mice and humans become increasingly frail with age, and we applied a recently validated mouse frailty index that permits the quantification of the accumulating deficits that occur with age and predicts mortality risk (Kane et al., 2016; Rockwood et al., 2017; Whitehead et al., 2014). We observed that both female and male *Rictor^Nkx2.1−/−^* mice develop significantly greater frailty than their control littermates (Figure 4a,b). As portended by this increased frailty, we find that loss of hypothalamic *Rictor* shortens the lifespan of both female and male mice (*p = *.005, log‐rank test stratified by genotype) (Figure 4c). Cox regression likewise indicated a significant negative effect of hypothalamic deletion of *Rictor* on survival (hazard rate (HR) = 1.69), and no interaction of genotype with sex was detected.

**Figure 5:**
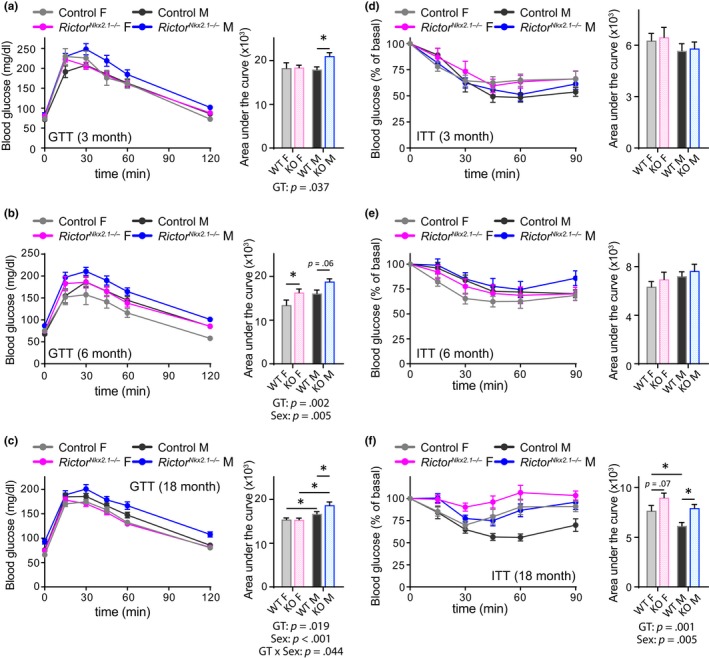
*Rictor^Nkx2.1−/−^* mice have lifelong impairment of glucose tolerance and develop insulin resistance. Metabolic health was assessed by performing (a–c) a fasting glucose tolerance test (GTT) and (d–f) a fasting insulin tolerance test (ITT) on both sexes of control and *Rictor^Nkx2.1−/−^* mice at approximately (a and d) 3 months, (b and e) 6 months, and (c and f) 18 months of age. (a and d) *n* = 6–14 mice/group, 2–3 months of age; (b and e) *n* = 9–10 mice/group, 5–6 months of age; (c and f) *n* = 20–32 mice/group, 15–20 months of age. Area under the curve: the overall effect of genotype (GT), sex, and the interaction represents the *p*‐value from a two‐way ANOVA; * = *p < *.05 from a Sidak's post‐test examining the effect of parameters identified as significant in the two‐way ANOVA. Error bars represent the *SEM*

Genetic disruption of mTORC2 signaling in several key metabolic tissues, including adipose tissue, liver, pancreas, and skeletal muscle, is associated with disruption of glucose intolerance and insulin resistance (Bentzinger et al., 2008; Blair, Archer, & Hand, 2013; Kumar et al., 2008, 2010; Lamming, Demirkan, et al., 2014; Lamming, Mihaylova, et al., 2014; Lamming et al., 2012; Polak et al., 2008; Tang et al., 2016). We found that both male and female *Rictor^Nkx2.1−/−^* mice exhibit lifelong mild glucose intolerance (statistically significant in males at all ages tested and in females only at 6 months of age, Figure 5a–c). Insulin sensitivity of young *Rictor^Nkx2.1−/−^* mice was similar to that of their littermate controls in both sexes (Figure 5d,e). However, *Rictor^Nkx2.1−/−^* mice developed age‐related insulin resistance, an effect that was particularly prominent in males (Figure 5f). Collectively, these results demonstrate a critical role for hypothalamic mTORC2 in maintaining physiological and metabolic health with age.

### 2.8 Loss of hypothalamic mTORC2 increases susceptibility to diet‐induced obesity

**Figure 6:**
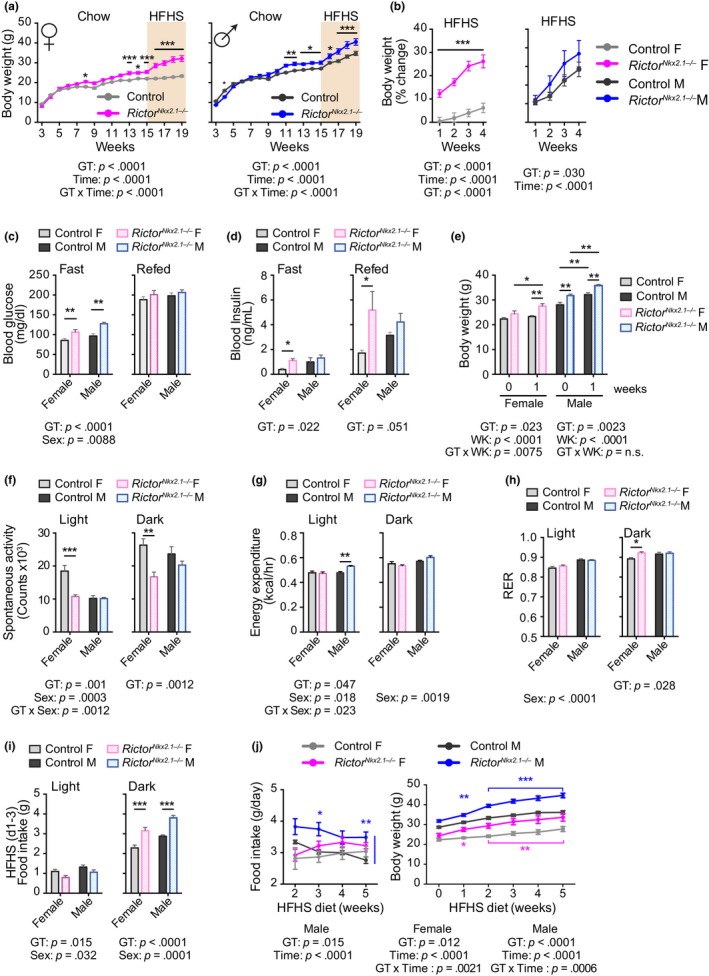
*Rictor^Nkx2.1−/−^* mice have increased susceptibility to diet‐induced obesity. (a and b) The body weight of control and *Rictor^Nkx2.1−/−^* mice of both sexes was tracked on chow diet and following a switch to a high‐fat, high‐sucrose (HFHS) diet as indicated, and (a) weight and (b) percentage weight gain on HFHS diet were plotted (*n* = 5‐12/group; Sidak test following two‐way ANOVA, * = *p < *.05, ** = *p < *.01, *** = *p < *.001). The overall effect of genotype (GT), time (T), and the interaction represents the *p*‐value from (a) a two‐way ANOVA or a (b) RM ANOVA. (c and d) Mice fed a HFHS diet for 3 weeks were fasted overnight and then refed for 4 hr, with collection of blood for the determination of (c) blood glucose and (d) insulin (*n* = 4–12 mice/group; Sidak's test following two‐way ANOVA, * = *p < *.05, **p = < .01). (e–i) Metabolic chambers were used to interrogate the metabolic effects of 1 week of HFHS diet feeding. (e) Body weight (f) spontaneous activity (g), energy expenditure per mouse (h) RER, and (i) average food intake during days 1–3 of HFHS feeding (*n* = 6 mice/group; Sidak's test following two‐way ANOVA, * = *p < *.05, ** = *p < *.01, *** = *p < *.001). (c–i) The overall effect of genotype (GT), sex, and the interaction represents the *p*‐value from a two‐way ANOVA. (j) Food intake (left) and body weight (right) of control and *Rictor^Nkx2.1−/−^* mice of both sexes was tracked on a HFHS diet (*n* = 6 mice/group; Sidak's test following RM two‐way ANOVA, * = *p < *.05, ** = *p < *.01, *** = *p < *.001, blue/pink stars indicate significant difference vs. male/female controls). The overall effect of genotype (GT), time on diet (T), and the interaction represents the *p*‐value from a two‐way ANOVA. Error bars represent the *SEM*

Deletion of *Rictor* in the whole brain or POMC neurons (Kocalis et al., 2014), or in Nkx2.1‐expressing neurons (this report), increases adiposity in male mice under chow feeding. However, the interactions of these genotypes with high calorie diets that are more relevant to current eating habits have not been investigated and no previous studies have included females. We therefore challenged *Rictor^Nkx2.1−/−^* mice with a high‐fat, high‐sucrose (HFHS) diet. Both sexes lacking hypothalamic *Rictor* exhibited increased susceptibility to diet‐induced obesity, with significantly greater weight gain in females detectable even during the first week (Figure 6a,b). Adipose mass was significantly increased in *Rictor^Nkx2.1−/−^* mice as compared to littermate controls fed the same HFHS diet (Figure S9a,b).

We found that fasting blood glucose was significantly increased in both *Rictor^Nkx2.1−/−^* males and females fed a HFHS diet (Figure 6c). Fasting and refed plasma insulin levels were increased in female *Rictor^Nkx2.1−/−^* on HFHS diet (Figure 6d). Consistently, *Rictor^Nkx2.1−/−^* mice fed a HFHS diet tended to have impaired glucose tolerance and insulin sensitivity as compared to littermate controls fed the same HFHS diet (Figures S9c–e). Plasma and liver triglycerides trended to increase in female *Rictor^Nkx2.1−/−^* mice fed a chow diet (Figure S9f). Although there was no change in triglyceride levels in the plasma or livers of *Rictor^Nkx2.1−/−^* mice of either sex on HFHS diet as compared to littermate controls, lipid content was elevated in the gastrocnemius muscles of female *Rictor^Nkx2.1−/−^* mice (Figure S9g–i). Thus, the lack of mTORC2 signaling in Nkx2.1 neurons exacerbates body weight gain and the resulting impairment of glucose homeostasis when mice are exposed to an unhealthy diet.

### 2.9 Hyperphagia is the primary driver of weight gain in *Rictor^Nkx2.1−/−^* mice

Because the body weights of *Rictor^Nkx2.1−/−^* mice remain stably elevated over those of controls across most of the lifespan, there is little opportunity to capture a substantial energetic imbalance. In order to gain insight into the mechanism that establishes this weight difference in *Rictor^Nkx2.1−/−^* mice, we placed mice in metabolic chambers during the first week of HFHS diet feeding, a period of rapid weight gain. As in the previous experiment, we observed that female *Rictor^Nkx2.1−/−^* mice gained significantly more weight than controls within one week of HFHS exposure (Figure 6e). Female *Rictor^Nkx2.1−/−^* mice maintained their low level of spontaneous activity following the shift from chow to HFHS diet (Figure 6f and Figure S5a). During the period of body weight gain on HFHS diet, there was no decrease in total energy expenditure on a per mouse basis or when adjusted for total body mass, suggesting that it could not account for the preferential weight gain of the *Rictor^Nkx2.1−/−^* mice (Figure 6g and Table S1). Female *Rictor^Nkx2.1−/−^* mice also exhibited a small but significant increase in RER (Figure 6h), suggesting a shift toward use of carbohydrates as an energy source and/or increased synthesis of fatty acids. Food intake was increased for the first three days during the dark period in both female and male *Rictor^Nkx2.1−/−^* mice (Figure 6i), and subsequently remained measurably higher in males but not in females during continued exposure to HFHS diet (Figure 6j). Thus, a modest increase in food consumption, rather than a decrease in energy expenditure, may be the primary driver of weight gain in *Rictor^Nkx2.1−/−^* mice.

## 3 DISCUSSION

The mTOR complexes are ancient sensors of nutrient status and metabolic state that have profound tissue‐specific effects on health and longevity. Inhibition of these complexes via rapamycin or genetic interventions that target mTORC1 signaling extends lifespan across species. Although the role of mTORC2 is comparatively less studied, targeting of this pathway in the liver is sufficient to shorten the lifespan of male mice, whereas disrupting mTORC2 in worms can alternately lead to increased or decreased longevity (Lamming, Mihaylova, et al., 2014; Mizunuma et al., 2014; Robida‐Stubbs et al., 2012; Soukas et al., 2009). Here, we report that hypothalamic mTORC2 activity increases with age in mice and that genetically ablating this complex in hypothalamic neurons is detrimental to metabolic health and longevity. *Rictor^Nkx2.1−/−^* mice are hypoactive, predisposed to adiposity and diet‐induced weight gain, become measurably more frail as they age, and have decreased overall survival.

The Nkx2.1 promoter drives expression of Cre recombinase in a wide range of hypothalamic nuclei (Ring & Zeltser, 2010; Xu et al., 2008). Reporter gene expression has also been mapped to scattered cells in the cerebral cortex, striatum, and globus pallidus, and in the thyroid, pituitary, and lung during development. Currently, there are no other genetic tools available that targets the majority of the neuronal subtypes that reside within the hypothalamus. The Nkx2.1‐Cre system has therefore been used widely to study the hypothalamus despite the limitations of its specificity (Burmeister et al., 2017; Chong, Greendyk, Greendyk, & Zeltser, 2015; Chong, Vogt, Vogt, Hill, Brüning, & Zeltser, 2015; Heinrich, Meece, Wardlaw, & Accili, 2014; Ring & Zeltser, 2010). We attempted to overcome this limitation using an inducible Nkx2.1.Cre model to delete *Rictor* during adulthood (Taniguchi et al., 2011). However, we did not observe a decrease in *Rictor* mRNA expression in the hypothalamus or difference in body weight and adiposity in this mouse model (data not shown). This is consistent with a prior report that *Nkx2.1* expression is substantially reduced after birth (Magno, Catanzariti, Nitsch, Krude, & Naumann, 2009). Thus, with currently available systems, we are unable to completely the avoid the potential of off‐target effects resulting from constitutive expression of *Nkx2.1‐Cre*. However, we note that thyroid hormone levels are not affected in *Rictor^Nkx2.1−/−^* mice, suggesting that the phenotypes we observe here do not result from inactivation of mTORC2 in the thyroid.

Both obesity and physical inactivity are thought to accelerate age‐related decline, either independently or in combination, and reduce life expectancy. While obesity per se is consistently related to all‐cause mortality across studies (Anon, 2016; Flegal, Kit, Orpana, & Graubard, 2013), it is increasingly appreciated that rapid weight gain early in life can be especially detrimental (Wagener, Müller, & Brockmann, 2013). Restricted *in utero* growth and/or transient lower body weight postnatally can trigger rapid catch‐up growth that is associated with shorter lifespan independently from adiposity (Hou, Bolt, & Bergman, 2011; Jennings, Ozanne, Dorling, & Hales, 1999; Ozanne & Hales, 2004; Ricklefs, 2006; Rollo, 2002; Sayer et al., 1998). We find that disruption of *Rictor* in hypothalamic neurons leads to lower body weight at the time of weaning followed by a rapid, excessive gain in body weight during postnatal development. In general, changes in food intake and/or energy expenditure were too modest to detect in young mice on chow diets. We view food intake as the most likely explanation for weight gain, given that subtle changes in food consumption are sufficient to explain considerable changes in body weight (Tschop et al., 2011), and that males are hyperphagic during refeeding. Moreover, food intake was clearly the major contributing factor in the accelerated weight gain experienced by both genders after switching to HFHS diet. We did, however, detect a modest decrease in energy expenditure in chow fed males at a single time point (10 months of age). Thus, it remains possible that there is also a small and potentially sex‐specific contribution of altered energy expenditure to the weight gain or higher weight maintenance of *Rictor^Nkx2.1−/−^* mice.

Weight gain in *Rictor^Nkx2.1−/−^* mice primarily reflected an increase in adiposity, yet we also observed a modest increase in circulating IGF‐1, femur length, and lean mass in the aging cohorts. Growth hormone‐releasing hormone, a neuropeptide expressed in the hypothalamus, stimulates the pituitary gland to release growth hormone, a major regulator of IGF‐1 expression (Junnila, List, Berryman, Murrey, & Kopchick, 2013). Thus, the increased levels of IGF‐1 that we observe could be due to altered hypothalamic release of growth hormone‐releasing hormone. Alternatively, they could also be a direct consequence of altered pituitary function. Further research will be required to distinguish between these possibilities and to determine the role of IGF‐1 in the metabolic effects we observed. Reduced signaling through the growth hormone/IGF‐1 axis due to genetic mutations or caloric restriction is associated with increased healthspan and lifespan in model organisms (Mao et al., 2018; Milman, Huffman, & Barzilai, 2016). These results support the idea that the early‐onset obesity observed in *Rictor^Nkx2.1−/−^* mice and the higher circulating level of IGF‐1 could have a combined long‐term negative impact on health and lifespan.

In addition, the majority of studies have found positive correlations between physical activity and longevity in rodents (Bronikowski et al., 2003; Holloszy, 1993; Holloszy, Smith, Vining, & Adams, 1985; Lokkegaard, Larsen, & Christensen, 2016; Mlekusch et al., 1996; Vogel et al., 2009) as well as humans (Lokkegaard et al., 2016; Rizzuto & Fratiglioni, 2014; Vogel et al., 2009). In normal weight individuals, regular physical activity has been estimated to extend life by 7.2 years, and conversely, inactivity to decrease life by 3.1 years (Moore et al., 2012). *Rictor^Nkx2.1−/−^* mice have substantially reduced spontaneous activity, and understanding the pathways that control this intrinsic drive to move could lead to new approaches to target a key modifiable factor that imparts resistance to stress and injury in older adults and delays the onset of age‐associated diseases (Cabanas‐Sánchez et al., 2018; Huffman, Schafer, & LeBrasseur, 2016).

Physical activity is an important component of energy expenditure in humans that negatively correlates with body weight gain and can act independently from changes in food intake (Bamman et al., 2014; Johannsen & Ravussin, 2008; Ladabaum, Mannalithara, Myer, & Singh, 2014; Mozaffarian, Hao, Rimm, Willett, & Hu, 2011; Pontzer et al., 2016; Warburton, Nicol, & Bredin, 2006). Thus, our finding that locomotor activity decreased in the absence of a measurable change in energy expenditure in young adult *Rictor^Nkx2.1−/−^* mice may appear counterintuitive. However, several recent studies have indicated that the effect of locomotor activity on total energy expenditure is far less in mice than in humans, and may be negligible under the conditions used for most experiments (Abreu‐Vieira, Xiao, Gavrilova, & Reitman, 2015; Dauncey & Brown, 1987; Moruppa, 1990; O'Neal, Friend, Guo, Hall, & Kravitz, 2017; Virtue, Even, & Vidal‐Puig, 2012). While past estimates have placed the fraction of total energy expenditure devoted to physical activity in mice as high as 38% (Dauncey & Brown, 1987), Virtue et al. (2012) have suggested that these studies overestimated the contribution of physical activity per se because other energy consuming processes correlate with activity. They determined that the true energetic cost of physical activity is ~10% of total energy expenditure at thermoneutrality, and much less under standard housing conditions, consistent with a prior study that used similar methods to estimate ~5% (Moruppa, 1990). Thus, even the profound decrease in locomotor activity that we observe in *Rictor^Nkx2.1−/−^* mice is likely to account for only a very small change in energy expenditure or weight gain. However, the neural pathways controlling the set point for activity level are of significant interest, given the clear benefits of both deliberate exercise and spontaneous movement for human health (Bamman et al., 2014; Johannsen & Ravussin, 2008; Pontzer et al., 2016; Warburton et al., 2006). Elevated home cage activity early in the dark period and during food deprivation can be associated with food seeking behavior, even when total food intake is unchanged (Mistlberger, 1994; Yang et al., 2015). To clarify whether motivation to obtain food was altered in the *Rictor^Nkx2.1−/−^* mice, we measured lever pressing in a progressive ratio operant task. The results clearly indicate that *Rictor^Nkx2.1−/−^* mice are equally motivated to obtain food under both ad libitum feeding and fasting conditions. Collectively, our findings support a direct regulation of physical activity level by neuronal mTORC2, rather than a secondary effect of food seeking behavior.

Mice lacking *Rictor* in Nkx2.1‐expressing cells display markedly increased susceptibility to diet‐induced obesity, a phenotype that was not previously assessed in mice lacking neuronal mTORC2 activity (Kocalis et al., 2014). Total energy expenditure was unaffected by genotype in mice consuming a high‐fat, high‐sucrose diet, suggesting that food intake (or absorption) plays a major role in weight gain. Consistently, higher food intake was recorded in both sexes over the first few days of HFHS diet feeding, when the rate of weight gain was highest, and food intake remained high in males over the subsequent weeks. Obesity and adiposity are well known to be associated with impaired glucose homeostasis, and thus, a limitation of the present study is that we cannot directly assess the direct versus indirect regulation of glucose homeostasis by hypothalamic mTORC2. We note that on chow diet, glucose intolerance is more prevalent in male *Rictor^Nkx2.1−/−^* mice, whereas the increase in adiposity is more pronounced in females, suggesting that the two effects may be somewhat independent. As we observed changes in the levels of several hypothalamic neuropeptides (e.g., AgRP, NPY, POMC, CART) involved in satiety, food intake, and energy balance, we consider it likely that the metabolic effects of hypothalamic *Rictor* on distinct neuronal populations mediate growth, adiposity, and metabolic phenotypes. A direct effect of hypothalamic *Rictor* loss on glucose tolerance would be consistent with the previously proposed role for central and hypothalamic insulin resistance in the maintenance of systemic glucose homeostasis (Chen, Balland, & Cowley, 2017; Koch et al., 2010).

It will be of significant interest to elucidate the molecular events that lie upstream and downstream of mTORC2 activity in hypothalamic neurons. Insulin signaling is known to stimulate mTORC2‐dependent phosphorylation of Akt S473 in multiple cell types, and neuron‐specific disruption of the insulin receptor (*IR*) driven by *Nestin‐Cre* increases body weight and fat mass (Bruning et al., 2000; Kappeler et al., 2008). However, deletion of the *IR* in Nkx2.1‐expressing neurons does not have any effect on body weight or composition (Chong, Greendyk, et al., 2015), possibly due to the IGF‐1 receptor playing a more prominent role than the *IR* in the hypothalamus (Kleinridders, Ferris, Cai, & Kahn, 2014) or due to activation of PI3‐kinase downstream of the leptin receptor (Lamming, 2014). Intriguingly, disruption of the IR in the arcuate nucleus reduces physical activity in young mice (Lin et al., 2010; Taguchi, Wartschow, & White, 2007), and re‐establishment of IR expression specifically in POMC neurons is sufficient to restore physical activity (Lin et al., 2010). These findings support the notion that the hypothalamic IR/mTORC2/Akt signaling cascade plays an important role in determining body weight homeostasis and locomotor activity in vivo. It is interesting to speculate that the age‐dependent increase of mTORC2 activity we observed in the hypothalamus of wild‐type mice may help to preserve fitness and longevity by promoting physical activity.

## 4 CONCLUSION

We demonstrate that hypothalamic mTORC2 signaling regulates physical activity independently from food seeking and is essential for normal metabolic health and longevity. In humans, it is well established that obesity correlates with physical inactivity and that exercise is a key modifiable lifestyle factor that improves overall health, decreases adiposity, and positively influences life expectancy (Goedecke & Micklesfield, 2014; Patterson & Levin, 2008; Verheggen et al., 2016). Thus, elucidation of the relevant neuronal populations, neural circuitry, and downstream signals responsible for mTORC2‐mediated changes in activity and energy balance has the potential to offer new strategies to treat obesity and extend healthspan that are complementary to existing strategies for diet modification. Moreover, our findings imply that mTOR kinase inhibitors now being explored as interventions to rejuvenate aged tissues in the elderly (Mannick et al., 2018), as well as mTORC2‐specific inhibitors in development for the treatment of cancers (Murray & Cameron, 2017), should be evaluated carefully for long‐term effects on frailty and general health.

## 5 MATERIALS AND METHODS

### 5.1 Materials

Antibodies to phospho‐Akt S473 (4060, 9271), phospho‐Akt T308 (9275), Akt (4691, 9272), Rictor (2140), phospho‐mTOR S2481 (2974), mTOR (2972), and phospho‐Stat3 Y705 (9145) were from Cell Signaling Technology. The β‐actin HRP (ab49900) antibody was from Abcam. Antibody to NeuN (MAB377) was from EMD Millipore Sigma. The Alexa Fluor 488 (A11029) and Alexa Fluor 594 (A11012) antibodies were purchased from Thermo Fisher Invitrogen. Vectashield Mounting Media with DAPI (H‐1200) was purchased from VWR. SignalStain Ab Diluent (8112) was purchased from Cell Signaling Technology. DAKO^®^ Protein Block Serum‐Free was purchased from Fisher Scientific. Tough Tubes with Caps (13119‐500) and 1.4‐mM ceramic beads (13113‐325) were purchased form Mo‐Bio Laboratories. Protease and phosphatase inhibitor cocktail tablets were from Thermo Fisher.

#### 5.1.1 Animal use and care

All animal procedures conducted at the University of Pennsylvania and the William S. Middleton Memorial VA Hospital were approved by the University of Pennsylvania Institutional Animal Care and Use Committee, and the Institutional Animal Care and Use Committee of the William S. Middleton Memorial Veterans Hospital, respectively. Mice were maintained under 12‐hr light/dark cycles at ~21°C and either fed a standard laboratory chow (U. Penn: Laboratory Rodent Diet 5010, LabDiet; Madison: Laboratory Rodent Diet 5001; LabDiet) or a high‐fat, high‐sucrose diet (Research Diets, D08112601, 45 kcal% fat and 30 kcal% sucrose). C57BL/6J.Nia mice obtained at different ages for Western blotting were maintained by the National Institute on Aging Aged Rodent Colony with ad libitum access to NIH 31 diet and were housed locally for 2–4 weeks with ad libitum access to LabDiet 5001 diet and free access to water prior to euthanasia by cervical dislocation. Mice were fasted overnight and sacrificed approximately 16 hr later as previously described (Baar, Carbajal, Ong, & Lamming, 2016). For immunohistochemistry, C57BL/6J.Nia female mice were obtained at approximately 4 months of age (young) and 16–19 months of age (old) and housed with ad libitum access to LabDiet 5001 diet and free access to water prior to perfusion as described below at approximately 6 months (young) or 22 months (old) of age. Mice lacking *Rictor* in *Nkx2.1*‐expressing neurons were generated by crossing *Rictor^fl/fl^* mice (Guertin et al., 2009) to mice expressing Cre recombinase under the control of the *Nkx2.1* promoter (Xu et al., 2008); mice were backcrossed to C57BL/6N and genotyped to ensure all mice expressed a functional copy of *Nnt* (Fontaine & Davis, 2016). Littermate *Rictor^fl/fl^* or pooled *Rictor^fl/fl^* and *Rictor^fl/+^* are defined as controls in the figures. For tissue harvest, mice were sacrificed by cervical dislocation and tissues were harvested and frozen in liquid nitrogen and stored at −80°C until use.

### 5.2 Immunoblotting

For Western blots in Figure S1a, mice were fasted overnight and sacrificed approximately 16 hr later. Cells and tissue samples were lysed in cold RIPA buffer supplemented with phosphatase inhibitor and protease inhibitor cocktail tablets. Tissues were lysed in RIPA buffer as previously described (Arriola Apelo et al., 2016) using a FastPrep 24 (M.P. Biomedicals) with bead‐beating tubes and ceramic beads (Mo‐Bio Laboratories), and then centrifuged for 10 min at 17, 000 × *g*. Protein concentration was determined by Bradford (Pierce Biotechnology). 20 µg protein was separated by SDS–PAGE (sodium dodecyl sulfate–polyacrylamide gel electrophoresis) on 8%, 10%, or 16% resolving gels (Life Technologies/Thermo Fisher) and transferred to PVDF membrane and then immunoprobed as previously described (Baar et al., 2016). Imaging was performed using a GE ImageQuant LAS 4000 imaging station, and images were quantified using NIH ImageJ. Samples in which no total protein was detected were excluded from the analysis of both the phosphorylated and total protein. For immunoblots in Figure 1d,e, and S1b whole cell lysates were prepared by homogenizing frozen tissue with RIPA lysis buffer supplemented with protease and phosphatase inhibitor cocktails (Roche) in TissueLyser (Qiagen). Twenty micrograms of whole cell lysate was run on 4%–15% gradient gel (Bio‐Rad) and transferred to PVDF membrane (Immobilon). Each blot was cut into maximum of three strips, blocked with 5% blotting blocker (Bio‐Rad), and probed with different primary antibodies (Rictor and phosphoproteins) at 1:2,000 dilution followed by secondary antibody incubation (1:5,000 dilution). Immunoblots were developed using SuperSignal West femto or pico kit (Thermo Fisher Scientific) on a Bio‐Rad Imaging System. Membranes were stripped and reprobed for total mTOR, Akt, and β‐actin.

### 5.3 Immunohistochemistry

C57BL/6J.Nia female mice were gravity perfused after an overnight fast (approximately 16 hr) through the left ventricle with 10% formalin for 15 min. Brains were removed, sliced in half in a sagittal plane on ice, and fixed in 10 ml 10% formalin overnight in 4°C. Subsequently, the tissue was transferred to 10 ml of 30% sucrose for 24–48 hr (until the tissue sank to the bottom of a 15 ml conical) at 4°C. Once fixed, the tissues were embedded in OCT compound and stored at −80°C until sectioning. Sectioning was done with a Lecia DM 4000B cryostat at 10 µm. Slides were washed with 1X PBS twice at RT. Blocking was done by incubating the slides in serum‐free blocking solution (Dako) for 30 min at RT. Slides were then washed three times with PBS. Primary antibodies targeting p‐Akt S473 (1:200 dilution) and NeuN (1:100 dilution) were used. Secondary antibodies targeting primary antibodies were conjugated with Alexa Fluor 488 (1:1,000 dilution) or Alexa Fluor 594 (1:1,000 dilution). DAPI (Thermo Scientific) was used to mount the slides and microscopy imaging was done using a Leica CM 1950. Images for each channel were obtained using a 20× objective, and scale bars were inserted manually by the investigator.

### 5.4 Metabolic studies

Multiple metabolic parameters including O_2_, CO_2_, food consumption, respiratory exchange ratio (RER), energy expenditure, and activity tracking were recorded using the Comprehensive Lab Animal Monitoring System (CLAMS, Columbus Instruments). For analysis of young 4‐week‐old female mice, mice were fed normal chow and housed in the metabolic chambers for 6 days. Data are represented as average values over the last 4 days. For longitudinal analysis of aging mice, mice were fed normal chow and acclimated to the metabolic chambers for approximately 24 hr prior to data collection, and data from a continuous 24 hr period were then selected for analysis as previously described (Yu et al., 2018). For analysis of diet‐induced obese mice, 6‐ to 8‐month‐old mice were singly housed for 4–7 days in home cages for acclimatization and then moved to the metabolic chambers and maintained for 6 days on normal chow, followed by a switch to HFHS diet for 7–8 days with ad libitum access to food and water. On normal chow, data for both male and female mice are represented as an average of 5 days (day 2 to day 6). For HFHS, data are represented as an average of all available days. For routine food intake measurements, mice were singly housed and food was weighed once or twice a week in the home cage.

### 5.5 Telemetry

Mice were implanted with telemetry transmitters (TA11PA‐F10, weight 1.6 g, Data Sciences International) in the peritoneal cavity for continuous monitoring of both core body temperature and home cage activity as described previously (Paschos et al., 2018; Yang et al., 2016). For telemetry monitoring, singly housed mice in home cages were placed in a ventilated, temperature‐controlled chamber with ad libitum access to food and water, with the exception of the indicated fasting period, during which they had access to water only.

### 5.6 Body composition

Body composition was measured by magnetic resonance imaging (EchoMRI, Echo Medical Systems).

### 5.7 Treadmill and running wheel

Treadmill performance was measured using an Exer3‐6 treadmill system (Columbus Instruments) as previously described (Frederick et al., 2015). Total distance run was defined as distance run before accumulation of 50 cumulative shock stimuli. Voluntary running was assessed using computer‐monitored running wheels (Columbus Instruments).

### 5.8 Frailty and longevity

Frailty and longevity were assessed in a total of 116 *Rictor^Nkx2.1−/−^* mice and wild‐type (*Rictor^fl/fl^*) littermates of both sexes (Table S2); two mice that died in close proximity to metabolic assessment were excluded from the analysis. Frailty was assessed longitudinally in a subset of mice starting at approximately 21 months of age using a 26‐item frailty index based on the procedures defined by Whitehead et al. (2014). The items scored included alopecia, loss of fur color, dermatitis, loss of whiskers, coat condition, tumors, distended abdomen, kyphosis, tail stiffening, gait disorders, tremor, body condition score, vestibular disturbance, cataracts, corneal opacity, eye discharge/swelling, microphthalmia, vision loss, menace reflex, nasal discharge, malocclusions, rectal prolapse, vaginal/uterine/penile prolapse, diarrhea, breathing rate/depth, and piloerection.

### 5.9 Operant task

The operant task experiment was performed in a conditioning chamber equipped with active (delivers food pellet) and inactive (does not deliver food pellet) levers as described previously (Alhadeff & Grill, 2014; Betley et al., 2015). Ad libitum fed mice were trained to perform the lever‐pressing task on a fixed ratio of one press per pellet (FR1) to obtain 20mg food pellets. This training was done overnight and followed sequentially by overnight training on FR3 and FR5 schedules. Following training, mice were tested on exponential progressive ratio (PR) schedule for either 1h during light period or overnight starting at dark period. The progressive ratio used followed the function of *F*i = 5e^(0.2i) − 5, where *F*i is the number of lever presses required to obtain the next pellet at i, the pellet number. Breakpoint analysis data are presented as number of pellets obtained before mice fail to press for a 10‐min period.

### 5.10 Determination of insulin and plasma metabolites

Plasma insulin was measured using mouse insulin ELISA kit (ALPCO) according to the manufacturer's instructions. Plasma and tissue triglycerides were measured using the Infinity Triglyceride Assay kit (Thermo Scientific; TR22421). Plasma free fatty acids were assayed using a free fatty acid (FFA) kit (Sigma‐Aldrich; MAK044) or HR Series NEFA‐HR(2) (Wako Diagnostics). Plasma leptin was measured using a leptin ELISA kit (EMD Millipore EZML‐82K). Plasma IGF‐1 was measured using a mouse IGF‐1 ELSIA kit (Crystal Chem, 80574). Plasma T4 (Cortez Diagnostics; 3149‐18) and corticosterone (Invitrogen; EIACORT) were measured using ELISA kits.

### 5.11 Glucose and insulin tolerance tests

Glucose tolerance tests were performed by fasting the mice overnight for 16 hr and then administering glucose (1 g/kg) intraperitoneally (Arriola Apelo et al., 2016; Fontana et al., 2016) or via oral gavage (2 g/kg). Insulin tolerance tests were performed by fasting mice for 5 hr or overnight for 16 hr, and then injecting human insulin intraperitoneally (0.75 IU/kg). Blood glucose was measured periodically for 2 hr after administration of dextrose or insulin using either a Bayer Contour blood glucose meter and test strips, or a OneTouch glucometer and test strips.

### 5.12 Gene expression

Total RNA was isolated from tissues using TRIzol reagent. RNA was reverse‐transcribed using High Capacity cDNA Reverse Transcription Kit (Thermo Fisher Scientific Cat# 4368814). qPCR was performed using an Applied Biosystems 7900HT system with SYBR green master mix (Applied Biosystems). Primer sequences used for qPCR were as follows: *Rictor*: F: ATGTGGCCAAATTGCAAGGAGTA, R: AACCCGGCTGCTCTTACTTCT; *Actb:* F: GGCTGTATTCCCCTCCATCG, R: CCAGTTGGTAACAATGCCATGT; *Lep:* F: CAGACAGAGCTGAGCACGAAA, R: CTGCACCCTATGTCACCATCA; *Tbp*: F: CCCCTTGTACCCTTCACCAAT, R: GAAGCTGCGGTACAATTCCAG. *Pomc*: F: CGCTCCTACTCTATGGAGCACTT, R: TCACCTACCAGCTCCCTCTTG; *Agrp*: F: AGGGCATCAGAAGGCCTGACCAGG, R: CATTGAAGAAGCGGCAGTAGCACGT; *Npy*: F: ACCAGGCAGAGATATGGCAAGA, R: GGACATTTTCTGTGCTTTCTCTCATTA; *Cart* F: CCCGAGCCCTGGACATCTA, R: GCTTCGATCTGCAACATAGCG; *Hcrt*: F: GCCGTCTCTACGAACTGTTGC, R: CGCTTTCCCAGAGTCAGGATA; and *Lepr*: F: AGCTAGGTGTAAACTGGGACA, R: GCAGAGGCGAATCATCTATGAC. *Rictor* and neuropeptide expression in the hypothalamus was normalized to *Actb*, and *LepB* expression in adipose tissues was normalized to *Tbp*.

### 5.13 Statistical analysis

Data are expressed as mean ± *SEM* Statistical analysis was conducted using Prism 7/8 (GraphPad Software). Significance was tested by Student's *t* test for two group comparisons or ANOVA followed by a Tukey or Sidak post hoc test as specified in the figure legends for comparisons of three or more groups. Survival analyses were conducted in R (version 3.5.0) using the “survival” package (version 2.38) (Therneau, 2015). Kaplan–Meir survival analysis was performed with log‐rank comparisons stratified by sex and genotype. Cox proportional hazards analysis was performed using sex and genotype as covariates. Alpha was set at 5% (*p < *.05 considered to be significant). The EE ANCOVA analysis done for this work was provided by the NIDDK Mouse Metabolic Phenotyping Centers (MMPC, www.mmpc.org) using their Energy Expenditure Analysis page (http://www.mmpc.org/shared/regression.aspx).

## CONFLICT OF INTEREST

D.W.L has received funding from and is a scientific advisory board member of, Aeonian Pharmaceuticals, which seeks to develop novel, selective mTOR inhibitors for the treatment of various diseases.
