# Supplementary figures and images for: Hypothalamic mTORC2 is essential for metabolic health and longevity
Source: Aging Cell. 2019 Aug 1;18(5):e13014. doi: 10.1111/acel.13014 (PMC6718533; doi:10.1111/acel.13014)

Supplementary Figure 1. Deletion of *Rictor* in *Nkx2.1* expressing neurons

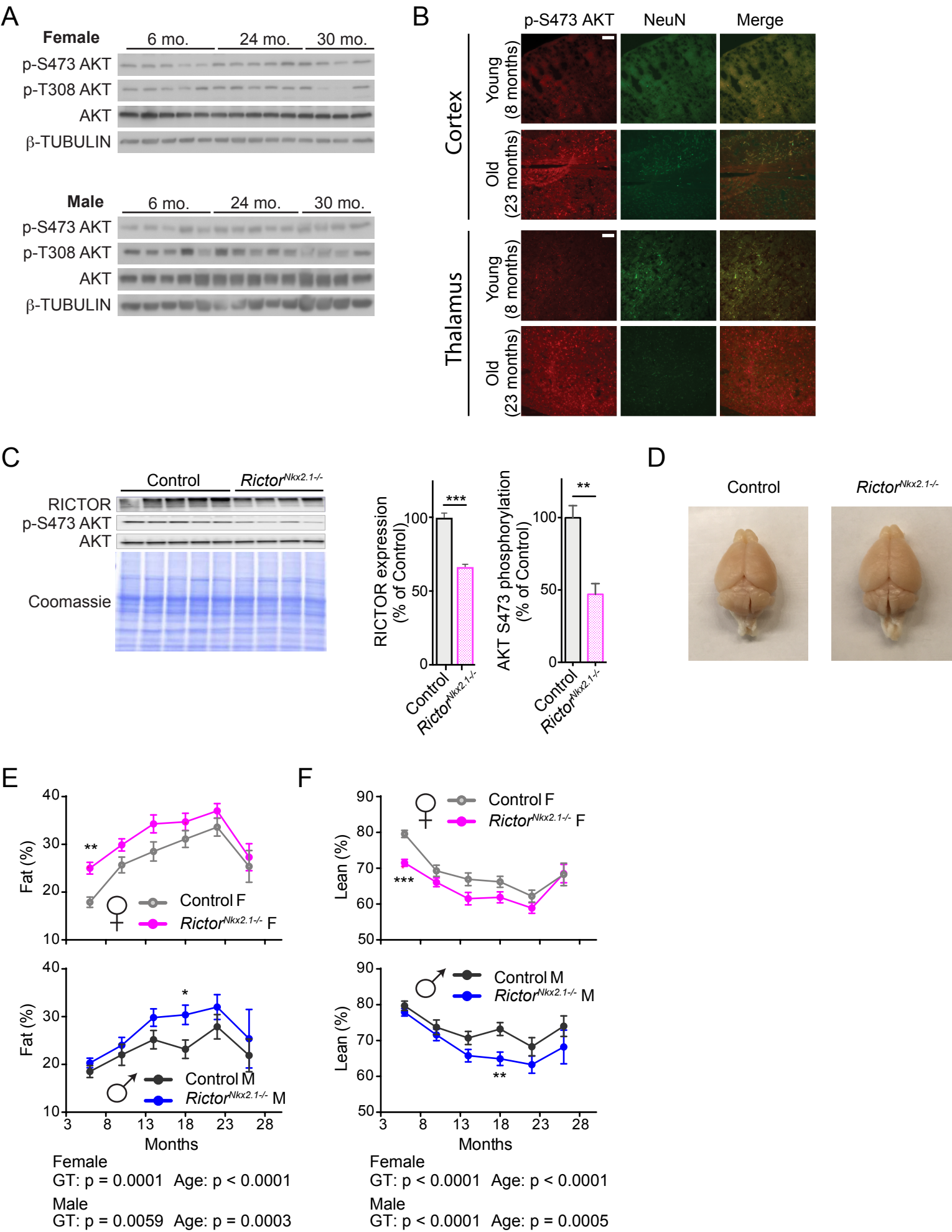

Supplement: Supplementary file 1 [file ACEL-18-e13014-s001.pdf]

Supplementary Figure 2. Characterization of young *Rictor<sup>Nkx2.1-/-</sup>* mice

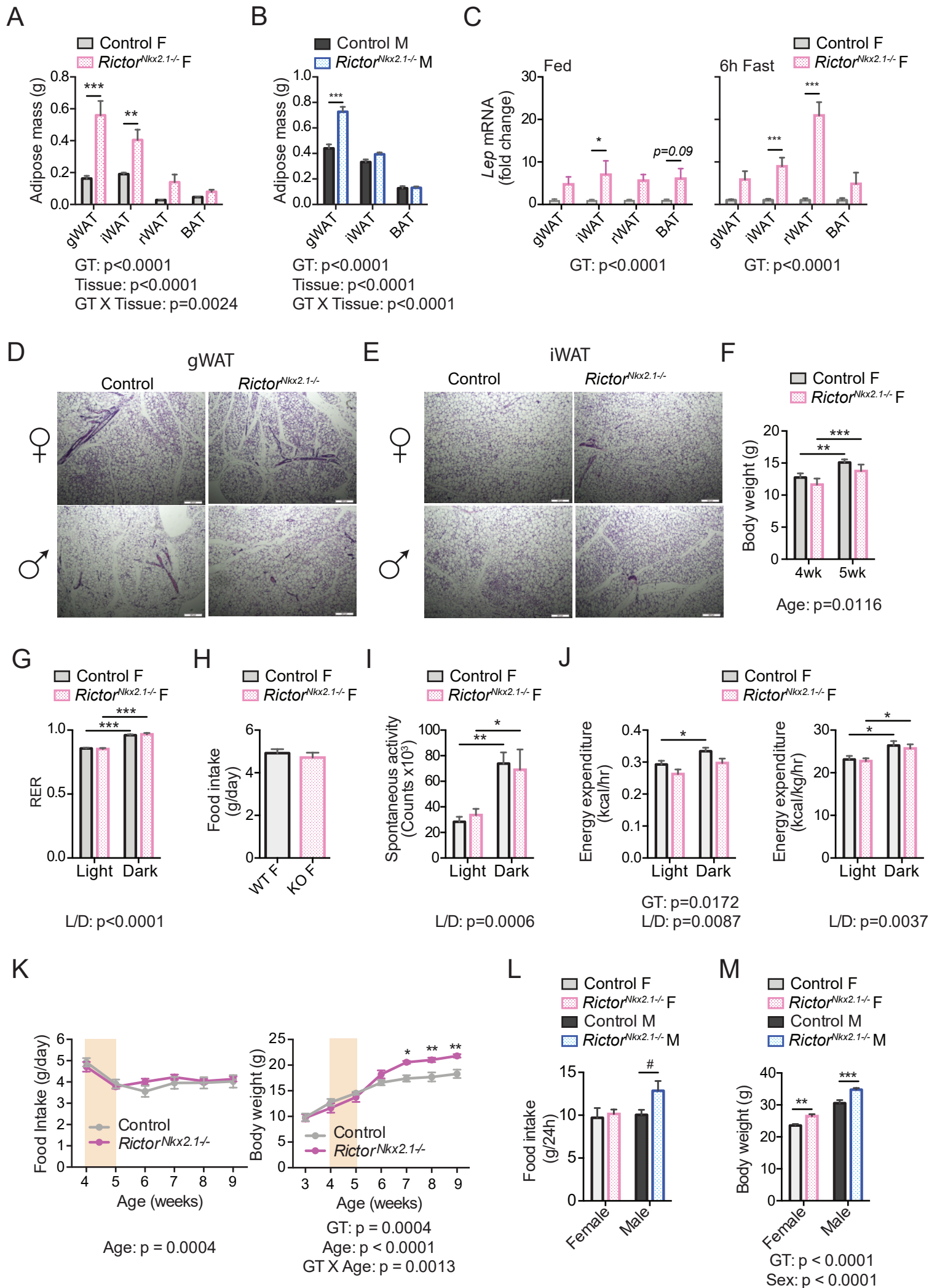

Supplement: Supplementary file 2 [file ACEL-18-e13014-s002.pdf]

Supplementary Figure 3. Positive energy balance in 10 month old male *Rictor*<sup>Nkx2.1-/-</sup> mice

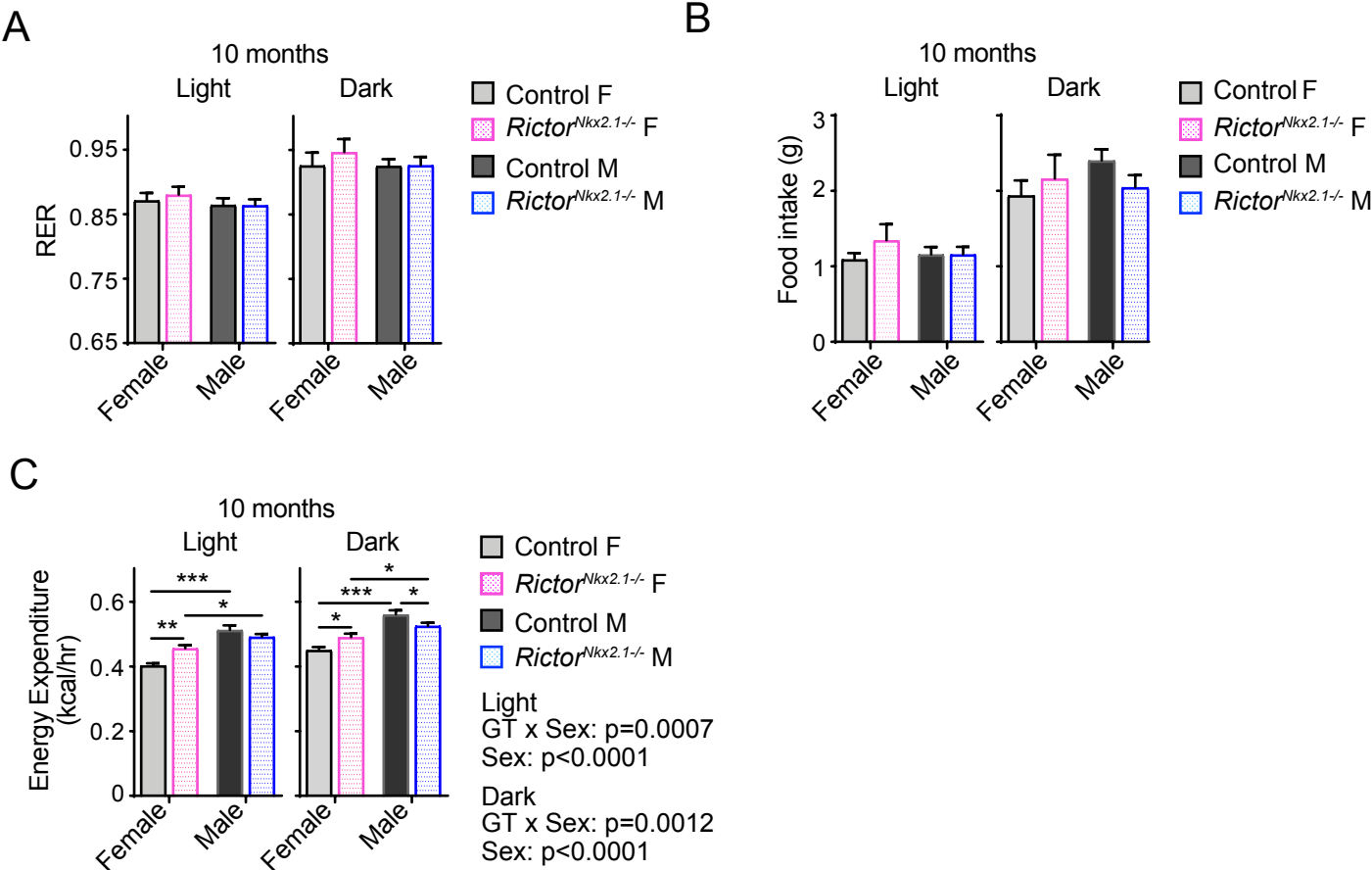

Supplement: Supplementary file 3 [file ACEL-18-e13014-s003.pdf]

Supplementary Figure 4. Unaltered energy balance in middle aged *Rictor<sup>Nkx2.1-/-</sup>* mice

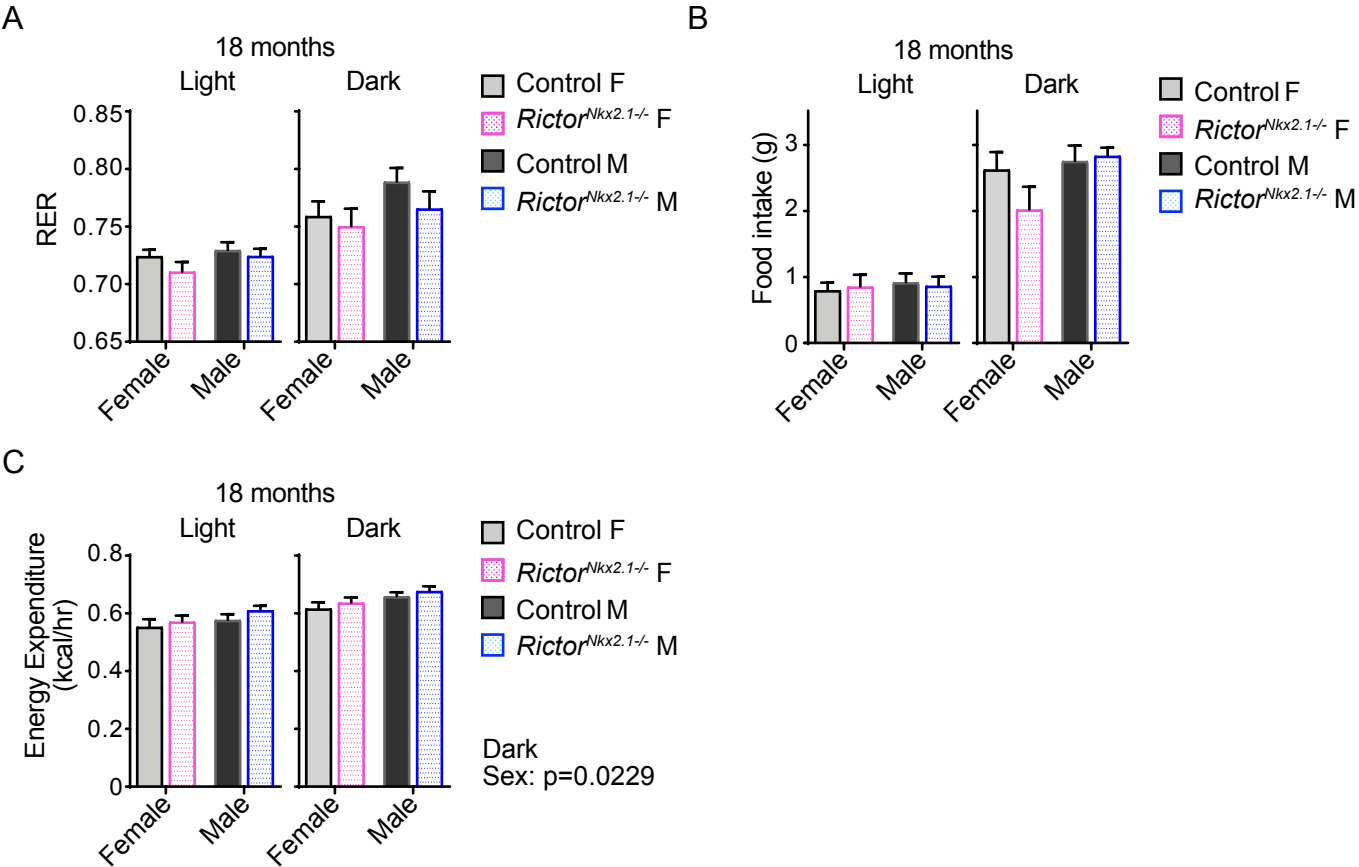

Supplement: Supplementary file 4 [file ACEL-18-e13014-s004.pdf]

Supplementary Figure 7. Hormonal changes in *Rictor*<sup>Nkx2.1-/-</sup> mice

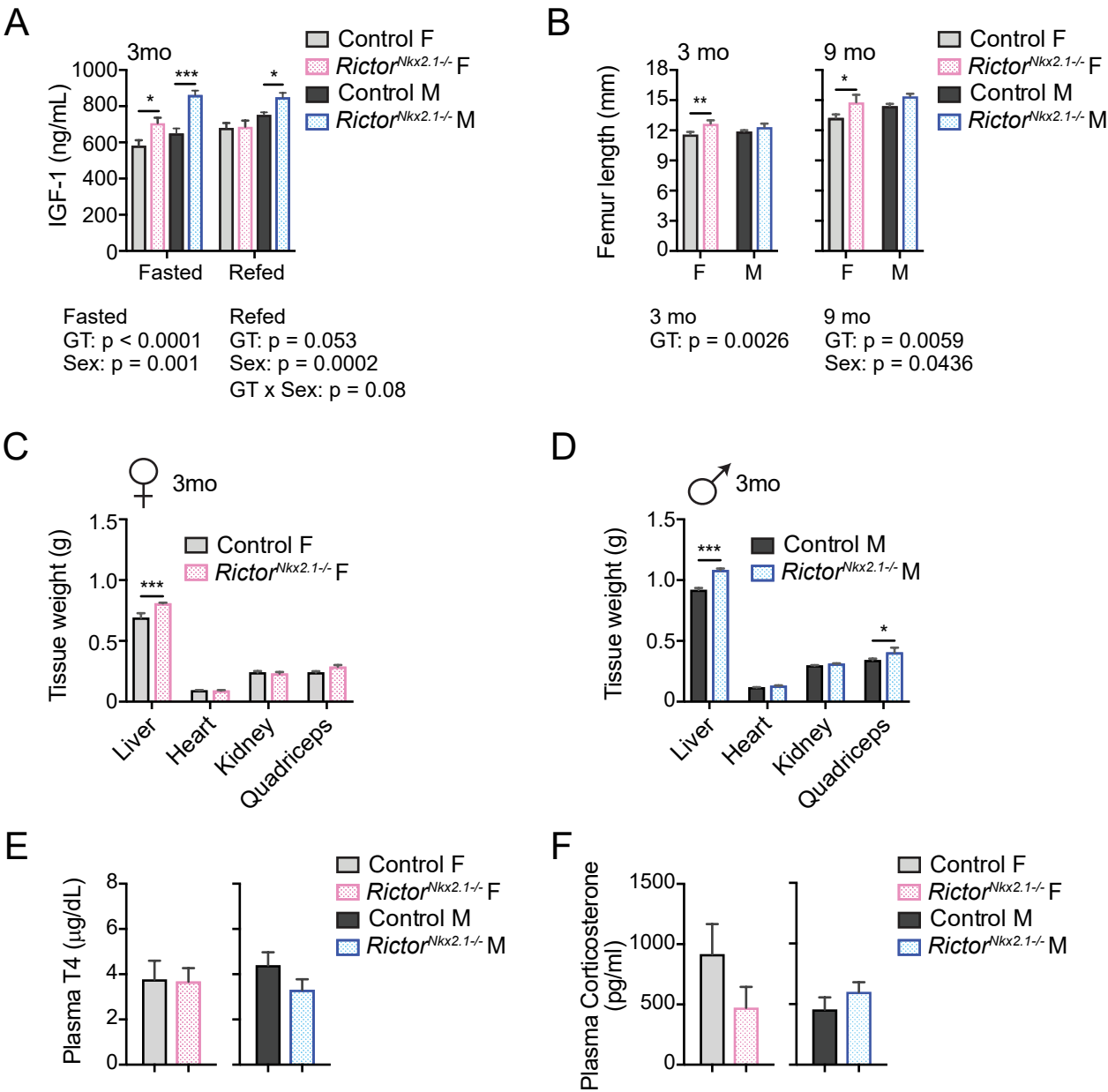

Supplement: Supplementary file 7 [file ACEL-18-e13014-s007.pdf]
