# Supplementary material for: Hypothalamic mTORC2 is essential for metabolic health and longevity
Source: Aging Cell. 2019 Aug 1;18(5):e13014. doi: 10.1111/acel.13014 (PMC6718533; doi:10.1111/acel.13014)

Supplementary Figure 5. Accelerated decline of sponataneous activity in *Rictor*$^{Nkx2.1-/-}$ knockout mice

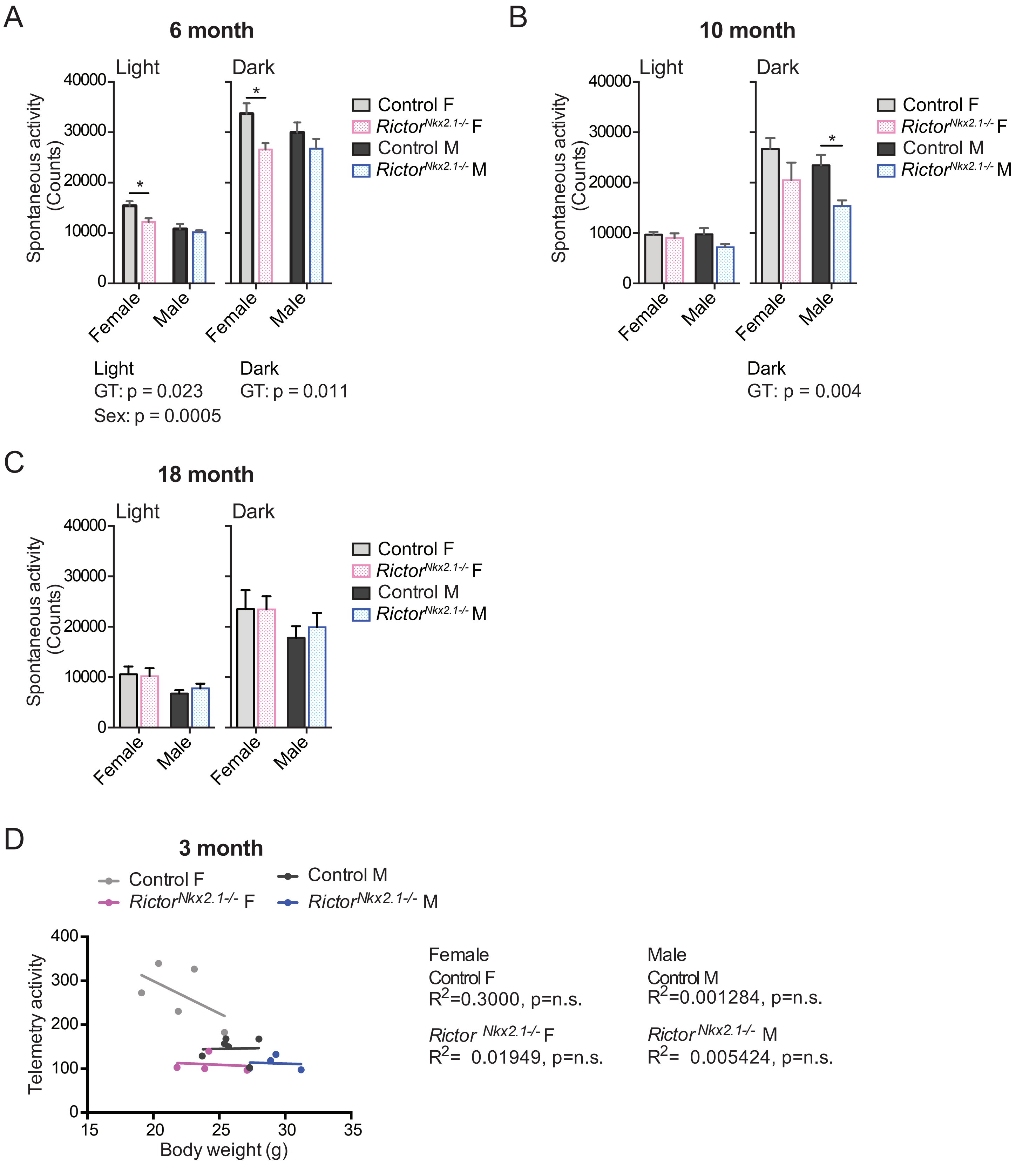

Supplement: Supplementary file 5 [file ACEL-18-e13014-s005.pdf]
