# Supplementary material for: Hypothalamic mTORC2 is essential for metabolic health and longevity
Source: Aging Cell. 2019 Aug 1;18(5):e13014. doi: 10.1111/acel.13014 (PMC6718533; doi:10.1111/acel.13014)
Supplement: Supplementary file 10 [file ACEL-18-e13014-s010.pdf]

Supplementary Table 1. Summary of ANCOVA analysis of energy expenditure in control and *Rictor*$^{Nkx2.1-/-}$ mice.

ANCOVA-adjusted group means, standard errors and significance

Responsible variable = Average Energy Expenditure (kcal/hr)
Covariate = Body mass (g)

Chow diet

Gender	Age		Control	Rictor$^{Nkx2.1-/-}$	P-Value
Female	4wk	Light	0.29 (0.007)	0.27 (0.008)	0.2278
		Dark	0.33 (0.007)	0.31 (0.008)	0.07652
	~6-7month	Light	0.42 (0.017)	0.35 (0.017)	0.0454
		Dark	0.51 (0.019)	0.42 (0.019)	0.02523
	10month	Light	0.41 (0.008)	0.45 (0.008)	0.003424
		Dark	0.46 (0.01)	0.48 (0.01)	0.08275
	18month	Light	0.56 (0.017)	0.57 (0.019)	0.5681
		Dark	0.62 (0.014)	0.65 (0.016)	0.1673

Male	~6-7month	Light	0.41 (0.032)	0.4 (0.032)	0.8383
		Dark	0.51 (0.044)	0.51 (0.044)	0.967
	10month	Light	0.52 (0.012)	0.49 (0.012)	0.1773
		Dark	0.56 (0.012)	0.52 (0.012)	0.04622
	18month	Light	0.59 (0.015)	0.6 (0.015)	0.8031
		Dark	0.67 (0.015)	0.67 (0.015)	0.955

HFHS diet

Gender	Age		Control	Rictor$^{Nkx2.1-/-}$	P-Value
Female	~6-7month	Light	0.48 (0.015)	0.47 (0.015)	0.6316
		Dark	0.55 (0.015)	0.53 (0.015)	0.4483
Male	~6-7month	Light	0.49 (0.01)	0.52 (0.01)	0.05668
		Dark	0.58 (0.013)	0.59 (0.013)	0.6189
