# Supplementary material for: Hypothalamic mTORC2 is essential for metabolic health and longevity
Source: Aging Cell. 2019 Aug 1;18(5):e13014. doi: 10.1111/acel.13014 (PMC6718533; doi:10.1111/acel.13014)
Supplement: Supplementary file 12 [file ACEL-18-e13014-s012.pdf]

Supplementary Figure Legends

Supplementary Figure 1. mTORC2 signaling in aged mice and the hypothalamus of *Rictor*$^{Nkx2.1-/-}$ mice.

(**A**) Whole brain lysates from female and male C57BL.6J.Nia mice at the indicated ages fasted overnight were examined by Western blotting to determine the phosphorylation of T308 and S473 of AKT, total AKT, and β-ACTIN. (**B**) mTORC2 activity, as determined by IHC-IF for phosphorylated AKT S473 (in red) in different brain regions of overnight fasted 23 month-old female C57BL.6J.Nia mice relative to young 8 month-old mice. A neuronal nuclei marker is targeted by the NeuN antibody (in green). Shown are representative images of cortex and thalamus regions (total n examined = 4 mice/group). Scale bar =100 µm. (**C**) Hypothalamic protein lysates from female Control and *Rictor*$^{Nkx2.1-/-}$ mice fasted for 6 hours were immunoblotted for RICTOR, phosphorylation of AKT S473, total AKT and coomassie. RICTOR expression was normalized to total protein loaded as assessed by coomassie staining, and phosphorylated AKT S473 is relative to total AKT. (**D**) Brains collected from 8 month old male Control and *Rictor*$^{Nkx2.1-/-}$ mice. (**E** and **F**) Longitudinal assessment of body composition, plotted as (**E**) percent fat mass and (**F**) percent lean mass of Control and *Rictor*$^{Nkx2.1-/-}$ mice (n= 5-29 mice/group; Holm-Sidak test following two-way ANOVA, * = p < 0.05, ** = p < 0.01, *** = p < 0.001). The overall effect of genotype (GT), Age, and the interaction represent the p-value from a two-way ANOVA. Error bars represent the SEM.

Supplementary Figure 2. Characterization of young *Rictor*$^{Nkx2.1-/-}$ mice.

(**A** and **B**) Weight of adipose tissue depots from (**A**) 12 week old females and (**B**) 24-26 week old male mice (n=5-9 mice per group; Sidak test following two-way ANOVA, * = p < 0.05, ** = p < 0.01, *** = p < 0.001). (**C**) Leptin mRNA was quantified in the indicated adipose tissues of female mice fed (left) or fasted for 6-8 hours (right) (n=4-7 mice per genotype; Sidak test following two-way ANOVA, * = p < 0.05, ** = p < 0.01, *** = p < 0.001). (**A**-**C**) The overall effect of genotype (GT), Tissue, and the interaction represent the p-value from a two-way ANOVA. (**D-E**) H&E stain of BAT (**E**) and gonadal adipose tissues (**F**) of five week old female and male mice. (**F-J**) Metabolic chamber measurements from 4 week old females: (**F**) body weight, (**G**) RER, (**H**) food intake, (**I**) spontaneous activity and (**J**) energy expenditure per mouse (left) or normalized to weight (right). (**K**) Food intake (left) and body weight (right) of female mice on normal chow. Colored region indicates period during which mice were in metabolic cage for data in figures S2F-S2J above. (**F-K**) n=4-5 mice per group; Sidak test following two-way ANOVA or two-way RM ANOVA, * = p < 0.05, ** = p < 0.01, *** = p < 0.001). The overall effect of either (**F-J**) genotype (GT) and Light/Dark (L/D), or (**K**) GT and Age, and the interaction represent the p-value from a two-way ANOVA or two-way RM ANOVA, respectively. (**L**) 24h refeeding food intake of 13-14 week old female and male mice following 24h food-deprivation (n=4-6 mice/group; uncorrected Fisher's LSD test following two-way ANOVA, # = p = 0.038). (**M**) Body weight of 26-33 week old females and males taken immediately prior to the metabolic chamber analysis presented in Figure 2H-2I. (n = 6/group, Sidak test following two-way ANOVA, ** = p < 0.01, *** = p < 0.001). The overall effect of genotype (GT) and Sex, and the interaction represent the p-value from a two-way ANOVA. Error bars represent the SEM.

Supplementary Figure 3. Positive energy balance in 10 month old male *Rictor*$^{Nkx2.1-/-}$ mice. (A-C) Metabolic chambers were used to assess (**A**) the respiratory exchange ratio (RER), (**B**) food consumption, and (**C**) energy expenditure per mouse by indirect calorimetry of 10 month old *Rictor*$^{Nkx2.1-/-}$ mice and Control littermates of both sexes (n = 8-10 mice/group; Holm-Sidak test following two-way ANOVA, * = p < 0.05, ** = p < 0.01, *** = p < 0.001). The overall effect of genotype (GT), Age, and the interaction represent the p-value from a two-way ANOVA. Error bars represent the SEM.

Supplementary Figure 4. Unaltered energy balance in middle-aged *Rictor*$^{Nkx2.1-/-}$ mice.

(**A-C**) Metabolic chambers were used to assess (**A**) the respiratory exchange ratio (RER), (**B**) food consumption, and (**C**) energy expenditure per mouse by indirect calorimetry of 18 month old *Rictor*$^{Nkx2.1-/-}$ mice and Control littermates of both sexes (n = (**A**) 8 mice/group, (**B**) 6 mice/group, (**C**) 8 mice/group; Holm-Sidak test following two-way ANOVA, * = p < 0.05). The overall effect of genotype (GT), Age, and the interaction represent the p-value from a two-way ANOVA. Error bars represent the SEM.

Supplementary Figure 5. Accelerated decline of spontaneous activity in *Rictor*$^{Nkx2.1-/-}$ mice

(**A-C**) Spontaneous activity of *Rictor*$^{Nkx2.1-/-}$ mice and Control littermates at (**A**) 6 months (**B**) 10 months and (**C**) 18 months of age (n = 6/group at 6 months, 8-10/group at 10 months, and 8/group at 18 months; Sidak test following two-way ANOVA, * = p < 0.05). (**D**) Linear regression analysis between body weight and telemetry activity of 14 week old females as in **Fig. 3A-3D**. Error bars represent the SEM.

Supplementary Figure 6. Reduced core body temperature in mice lacking *Rictor* in the hypothalamic neurons

(**A** and **B**) Traces of average core temperature of 13-14-wk old female (A) and male (B) mice under conditions indicated, as determined by telemetry with readings binned into 10-minute blocks. (**C** and **D**) Quantification of the data under fed condition is represented as an average of 2 days and during fasting and refeeding over ~24h time period (n=4-5 mice/group; Sidak test following two-way RM ANOVA or two-way ANOVA). The overall effect of genotype (GT), time of the day, and the interaction represent the p-value from (**A** and **B**) a two-way RM ANOVA or (**C** and **D**) a two-way ANOVA. Error bars represent the SEM.

Supplementary Figure 7. Hormonal changes in *Rictor*$^{Nkx2.1-/-}$ mice

(**A**) Plasma IGF-1 levels in 3 month old male and female *Rictor*$^{Nkx2.1-/-}$ mice (n=4-6 per group; Sidak test following two-way ANOVA, * = p < 0.05, *** = p < 0.001). (**B**) Femur length in three month (left) and nine month (right) old mice. (n=3-11 per group; Sidak test following two-way ANOVA, * = p < 0.05, ** = p < 0.01). (**C-D**) Lean tissue weights in three-

month-old females (**C**) and males (**D**) (n=3-11 per group; Sidak test following two-way ANOVA, * = p < 0.05, *** = p < 0.001). (**E**) Plasma T4 level in 12-week-old females (left) and 20-week-old males (right). (**F**) Plasma corticosterone level in in 12-week-old females (left) and 20-week-old males (right) (n= 4-9 mice per genotype. Student's *t*-test for control and KO comparisons). (**A-B**) The overall effect of genotype (GT), Age, and the interaction represent the p-value from a two-way ANOVA. Error bars represent the SEM.

Supplementary Figure 8. Leptin signaling and neuropeptide expression in $\textit{Rictor}^{Nkx2.1-/-}$ mice

(**A-B**) Percent body weight change (**A**) and food intake (**B**) in 18-week-old male controls and $\textit{Rictor}^{Nkx2.1-/-}$ mice 24h after saline and leptin injection. (n=6-9 per group; Sidak test following two-way ANOVA, * = p < 0.05, ** = p < 0.01 and *** = p < 0.001). (**C-D**) Hypothalamic protein lysates from 6h fasted females (**C**) and males maintained under thermoneutral condition for 9 days (**D**) were immunoblotted for phosphorylation of STAT3 Y705 and total STAT3. (**E-F**) Hypothalamic expression of neuropeptides in 10-12 week old female mice (**E**) and 24-26 week old male mice (**F**) under ad-libitum fed condition. (n= 4-9 mice per genotype. ***P<0.001, **P<0.01, *P<0.05, #P=0.09, control vs $\textit{Rictor}^{Nkx2.1-/-}$ mice). Error bars represent the SEM.

Supplementary Figure 9. Impaired metabolic homeostasis in $\textit{Rictor}^{Nkx2.1-/-}$ mice on high fat high sucrose diet

(**A** and **B**) Weight of adipose tissue from the indicated depots of (**A**) female and (**B**) male mice after 5 weeks on a HFHS diet. (n=5-12 mice/group; Sidak test following two-way ANOVA, * = p < 0.05, *** = p < 0.001). The overall effect of genotype (GT), Tissue, and the interaction represent the p-value from a two-way ANOVA. (**C**-**E**) Glucoregulatory control was assessed in $\textit{Rictor}^{Nkx2.1-/-}$ mice and Control littermates after 3-4 weeks of HFHS feeding by means of (**C**) an oral glucose tolerance test, (**D**) HOMA2-IR calculation, and an (**E**) insulin tolerance test (n=4-12 mice/group (GTT), 5-8 mice/group (HOMA2-IR), and 4 mice/group (ITT); Sidak test following two-way ANOVA, * = p < 0.05). The overall effect of genotype (GT) and sex, and the interaction represent the p-value from a two-way ANOVA. (**F**) Plasma (left) and liver triglyceride (right) level in chow fed 12-week old females (n=6-8 mice/group; Student's *t*-test for control and KO comparisons, # = p < 0.1). (**G-I**) Plasma (**G**), liver (**H**) and muscle (**I**) triglyceride levels of female and male mice on HFHS diet for 4-6-weeks. (n=5-12 mice/group; Sidak test following two-way ANOVA, *** = p < 0.0001). Error bars represent the SEM.

Supplementary Table Legends

Supplementary Table 1.

Summary of ANCOVA analysis of energy expenditure in control and $\textit{Rictor}^{Nkx2.1-/-}$ mice.

Supplementary Table 2.

Raw lifespan data for Figure 4C.
